# Programming Degradation and Drug Release Through Micropatterning of PLGA Films

**DOI:** 10.1021/acsami.6c04044

**Published:** 2026-05-13

**Authors:** Irene Guerriero, Cristiano Pesce, Raffaele Spanò, Stefania Sganga, Nicola Tirelli, Anna Lisa Palange, Daniele Di Mascolo, Paolo Decuzzi

**Affiliations:** † Laboratory of Nanotechnology for Precision Medicine, 121451 Fondazione Istituto Italiano di Tecnologia, 16163 Genoa, Italy; ‡ Department of Informatics, Bioengineering, Robotics and System Engineering, Università di Genova, 16145 Genoa, Italy; § Department of Pharmaceutical and Pharmacological Sciences, University of Padua, 35122 Padova, Italy; ∥ Laboratory of Polymers and Biomaterials, Fondazione Istituto Italiano di Tecnologia, 16163 Genoa, Italy; ⊥ Department of Electrical and Information Engineering, 18951 Politecnico di Bari, 70126 Bari, Italy; # Division of Oncology, Department of Medicine and Department of Pathology, Stanford University School of Medicine, Stanford, California 94305, United States

**Keywords:** degradation, erosion, drug diffusion, thin film, microgeometry

## Abstract

Polymeric films enable spatial and temporal control of drug release, improving therapeutic efficacy while reducing systemic side effects. While most studies focus on polymer blends, copolymer design, or chemical modification, this work investigates how geometry alone regulates degradation and drug release in micropatterned films. To this end, we exploit the μMESH platform, a dual-compartmentalized film composed of regularly patterned poly­(lactic-*co*-glycolic acid) (PLGA) microfilaments arranged to form square openings deposited over a poly­(vinyl alcohol) (PVA) microlayer. Four μMESH films with square openings of 5, 10, 20, and 50 μm were fabricated, along with a conventional unpatterned, solid PLGA film (FLAT). These films exhibited different surface area-to-volume ratios (*S*_*a*_/*V*), ranging from 0.40 μm^–1^ (FLAT) to 1.02 μm^–1^ (μMESH with 20 μm openings). After extensive microscopy characterizations, PLGA mass loss (erosion) and molecular weight reduction (degradation) were evaluated for each film over several weeks in different media. Erosion and degradation rates strongly correlated with *S*_*a*_/*V* (r = 0.99 and r = 0.92, respectively), with higher *S*_*a*_/*V* resulting in slower mass loss and molecular weight decay. Electron microscopy analyses confirmed prolonged structural retention in high *S*_*a*_/*V* films, with the μMESH exhibiting 20 μm openings preserving its architecture for at least 60 days, whereas lower *S*_*a*_/*V* films (FLAT) showed heterogeneous degradation as early as 14 days. These observations were qualitatively confirmed by in vivo studies. From a pharmacological perspective, docetaxel-loaded films exhibited biphasic release profiles dominated by diffusion, with cumulative drug release increasing with *S*_*a*_/*V* (r = 0.97). Overall, these findings demonstrate that the degradation, erosion, and drug release kinetics of PLGA films can be precisely tuned through geometry alone, providing a robust strategy for controlling the performance of implantable polymeric films.

## Graphical abstract

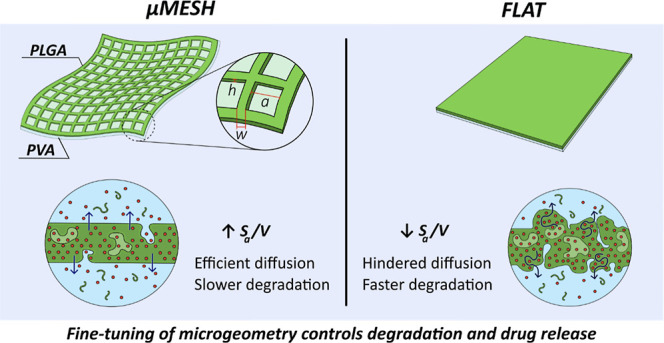

## Introduction

There is growing interest in developing drug delivery implants because of their ability to release a broad range of therapeutic agents, including small molecules, inhibitors, peptides, enzymes, and antibodies, in a controlled and sustained manner. Drug-loaded implants improve targeted delivery to specific tissues or organs, enhance drug penetration, reduce the required dosage, minimize systemic toxicity, and ultimately boost therapeutic efficacy.^1–3^ Among the many biomaterials explored, polymers are widely used due to their tunable physicochemical properties and biocompatibility. Natural polymers, such as collagen, gelatin, alginate, chitosan, and hyaluronic acid, are valued for their intrinsic bioactivity and ability to mimic extracellular matrix components, whereas synthetic polymers, including poly­(lactic acid) (PLA), poly­(lactic-*co*-glycolic acid) (PLGA), poly­(ε-caprolactone) (PCL), polyethylene glycol (PEG), and polyanhydrides, offer greater reproducibility and precise control over mechanical properties, degradation kinetics, and drug release profiles.^1,4^ Among these materials, poly­(lactic-*co*-glycolic acid) (PLGA) is a copolymer incorporated into numerous FDA-approved products for human use.^5,6^ A key advantage of PLGA is its ability to undergo hydrolytic degradation in physiologically relevant environments, resulting in the formation of lactic acid (LA) and glycolic acid (GA),^7^ which are biocompatible and eliminated through natural metabolic pathways.^6,8^

Hydrolytic degradation begins once water molecules penetrate the polymer matrix and proceeds through two primary mechanisms: bulk erosion and surface erosion. In surface erosion, water permeation in the polymer matrix is slower than hydrolysis at the surface, confining degradation to the outer layer and maintaining matrix integrity over extended periods. In contrast, during bulk erosion, water diffusion throughout the matrix is faster than hydrolysis, leading to polymer chain scission across the bulk.^8,9^ As degradation progresses and the molecular weight of the polymer chains decreases, soluble oligomers and monomers diffuse out, leading to mass loss (erosion).^10,11^ Importantly, the retention of acidic degradation byproducts within the polymer matrix can locally reduce pH and accelerate hydrolysis through an autocatalytic effect.^12^ For PLGA specifically, degradation kinetics depend on the initial molecular weight of the polymer, the length and ratio of LA and GA segments, as well as environmental factors such as pH, temperature, and the buffering capacity of the surrounding medium.^13^

Beyond the intrinsic physicochemical properties of the polymer, the geometry of the drug delivery implant also plays a crucial role, as the surface area-to-volume ratio (*S*_*a*_/*V*) strongly influences diffusion-limited processes such as hydrolysis.^3^ Numerous studies show that altering the dimensions of PLGA micro- and nanoparticles modulates degradation rates, with larger structures exhibiting faster bulk degradation due to restricted diffusion of acidic byproducts and, consequently, enhances autocatalysis.^14^ When drugs are incorporated into PLGA-based implants, release is typically governed initially by diffusion and subsequently by matrix degradation.^3,9^ Drug diffusion occurs concurrently with the outward transport of acidic degradation products and the inward diffusion of bases from the surrounding medium, indicating that the physicochemical properties of the drug can influence both release profiles and polymer degradation.^15^ Because diffusion depends on the effective pathway length, the geometrical features of the delivery platform are critical determinants of release kinetics.

Within this context, the biodegradable μMESH platform offers a unique opportunity to fabricate thin PLGA films with a precisely defined geometry (micropatterning) and to investigate its effect on degradation and pharmacological behavior. μMESH is a dual-compartmentalized polymeric implant composed of a PVA microlayer integrated with a PLGA micronetwork. Thanks to the modularity of the fabrication process, μMESH can be engineered with different geometries, enabling the tailoring of the physicochemical and pharmacological properties for specific applications. In this study, four distinct μMESH geometries were fabricated and compared with each other and with a continuous, nonpatterned PLGA film (FLAT) to evaluate how microarchitecture influences erosion, degradation, and drug release rates. Docetaxel (DTX) was selected as a model drug and loaded in the PLGA film for all configurations. Following extensive in vitro characterizations, the PLGA film with the slowest degradation and erosion rates was further evaluated in vivo and compared with the FLAT control. Morphological changes were assessed at multiple time points to determine whether exposure to physiological fluids accelerates or decelerates erosion.

## Materials and Methods

### Materials

Poly­(D,l-lactide-*co*-glycolide) (lactide/glycolide 50:50, Mn 38–54 kDa), poly­(vinyl alcohol) (PVA, Mw 9–10 kDa, 80% hydrolyzed), sodium azide, Gill No.2 Haematoxylin solution (Sigma-Aldrich, GHS232) and Eosin Y alcoholic solution (Sigma-Aldrich, HT110132), sucrose (Sigma-Aldrich, S7903) were purchased from Merck (Darmstadt, Germany). Poly­(dimethylsiloxane) (PDMS) Silgard 184 was obtained from Dow Corning (Germany). Curcumin (95% total curcuminoid content) from Turmeric rhizome and trifluoroacetic acid (TFA) 99% were purchased from Alfa Aesar GmbH & Co KG (Karlsruhe, Germany). Docetaxel (DTX, 99%) was purchased from Thermo Fisher Scientific (Segrate, Italy). Paraformaldehyde solution 4% in PBS (product code sc-281692) was from Santa Cruz Biotechnology Inc. (Heidelberg, DE). Surgipath FSC 22 Frozen Section Compound was purchased from Leica Microsystems GmbH (Wetzlar, DE).

### Fabrication of Micropatterned Polymeric Films

μMESH was prepared following a soft lithographic method, as previously described by the authors.^16^ Briefly, the main geometric features of μMESH were defined by direct laser writing, which induced local polymerization of a photoresist deposited on a silicon wafer. This was followed by reactive ion etching to transfer the pattern into the wafer, and a final rinsing and thermal baking step (Figure 1A). Specifically, a regular array of square pillars with edge lengths of 5, 10, 20, and 50 μm was defined. The channel width varied accordingly with the pillar size, while the depth remained constant (Figure 1E). It is noteworthy that all the geometric parameters can be easily modified by reprogramming the direct laser writing steps. This micropatterned geometry of the silicon master template was replicated using a molding process. First, a PDMS layer was obtained by pouring the polymeric base and the curing agent (10:1 ratio) onto the micropatterned silicon master and drying the mixture at 60 °C for 4 h. Then, the PDMS template was used to replicate the geometry further into a PVA microlayer, obtained by pouring the polymeric solution over the PDMS template and curing it at 60 °C for 2 h. Upon complete water evaporation, the resulting PVA microlayer was peeled off the PDMS template. The PVA microlayer retains the identical geometrical pattern as the original silicon template. The resulting channels (ridges) were filled with a polymeric paste made by dissolving known amounts of PLGA in acetonitrile (ACN). The resulting 30 × 30 mm composite film comprises the PVA microlayer (hydrophilic compartment) and the PLGA micronetwork (hydrophobic compartment). This composite film was then cut into multiple 5 × 5 mm pieces, referred to as μMESH. Additionally, a composite film without any geometric pattern was fabricated by spreading the PLGA polymeric paste over a flat PVA layer, resulting in a bilayer composite film referred to as FLAT.

**Figure 1:**
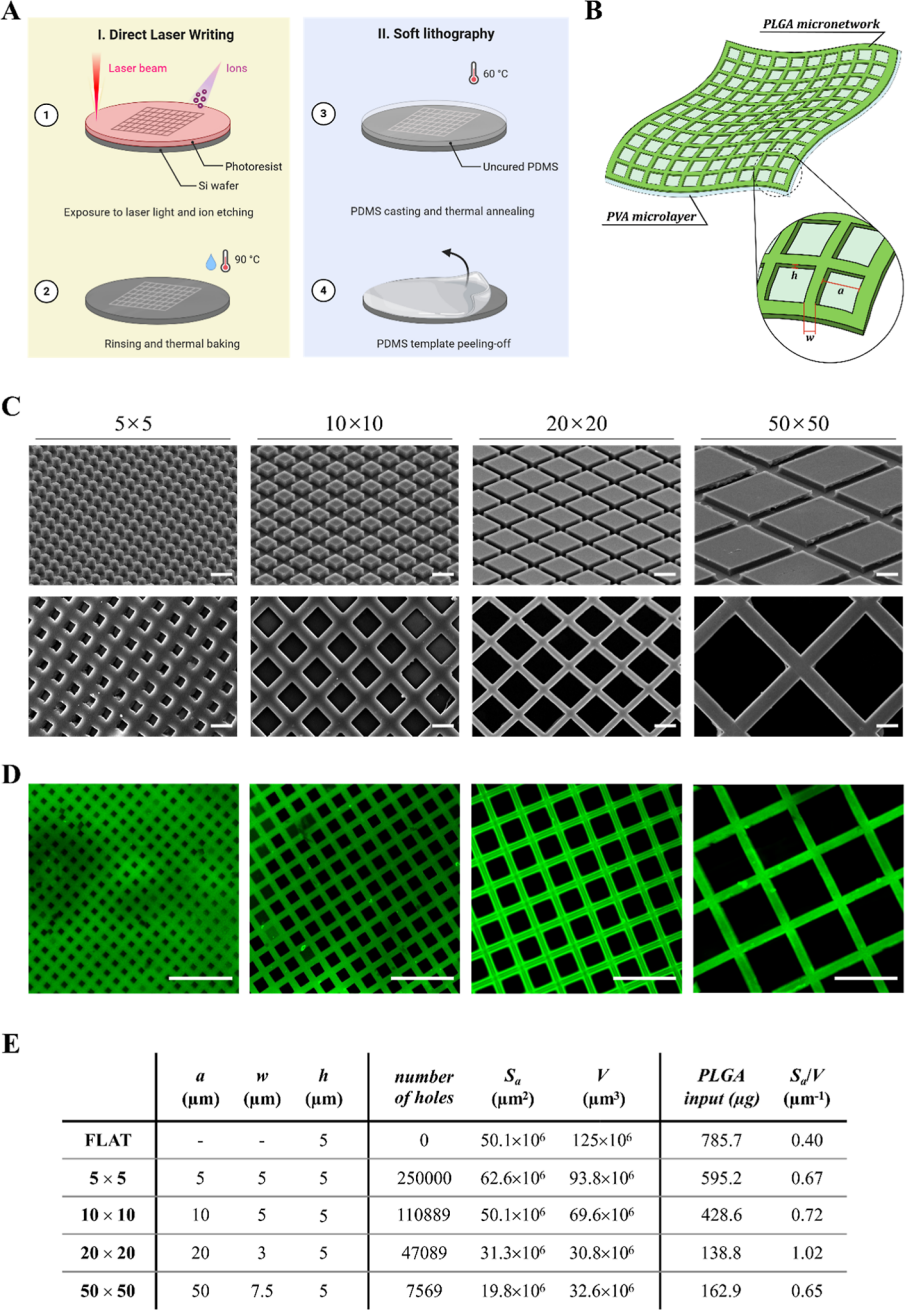
Fabrication and geometrical characterization of μMESH micropatterned PLGA films. (A) Schematic representation of the top-down fabrication process of μMESH (created in BioRender). (B) Schematic representation of μMESH with the inset highlighting the geometrical attributes of the micropatterned PLGA films. (C) Scanning electron microscopy images of the PVA microlayer (***first row***) and the PLGA micronetwork (***second row***), after PVA dissolution in aqueous solution (scale bar: 10 μm). (D) Confocal fluorescent microscopy images of the micropatterned PLGA film after PVA dissolution (scale bar: 50 μm). (E) Table listing the geometrical properties of four micropatterned PLGA films, originated from 5 × 5, 10 × 10, 20 × 20, and 50 × 50 μMESH, and the nonpatterned FLAT control (5 × 5 mm).

Hydrophobic molecules were easily incorporated into the PLGA paste. Specifically, 500 μg of green, fluorescent curcumin (CURC) was added per template to qualitatively assess loading efficiency and correct formation of the hydrophobic micronetwork by confocal microscopy (A1^+^/A1R^+^, Nikon Corporation, Tokyo, Japan).

### Degradation of Micropatterned Polymeric Films

To evaluate μMESH degradation over time, the PLGA micronetwork was first separated from the PVA microlayer. Note that given the molecular weight (9–10 kDa) and degree of hydrolysis (80%) considered in this work, the PVA microlayer dissolves rapidly following exposure to a physiological solution. Therefore, the dual-compartmentalized μMESH was placed into a Petri dish with the PVA component facing downward. Then, DI water was dropped on the air-facing side. After 2 h at room temperature (RT), the remaining PLGA micronetwork was gently washed to ensure full detachment from the substrate. The samples were swabbed with blotting paper, transferred onto a nylon sheet, and dried at RT.

The collected PLGA micronetworks were placed inside a sealed jar filled with 50 mL of DI water or 0.1 M PBS pH 7.4 containing 0.02% sodium azide. The number of micronetworks was adjusted to ensure that all the jars contained the same initial amount of PLGA (15 ± 0.05 mg). The jars were placed in an incubator at 37.0 ± 0.1 °C, and samples were taken at predetermined time points to monitor degradation, namely 7, 14, 21, 28, and 60 days. The incubation media were not replaced during the study to avoid affecting the erosion mechanisms.

### Mass-Loss Measurements

Samples were weighed before being placed in the degradation medium to determine the initial mass of the PLGA micronetwork, *m*_*ini*_. After fixed time intervals, the PLGA micronetworks were recovered by filtration using a nylon sheet with a pore size of 40 μm (pluriSelect life science UG & Co. KG, Germany). The samples were gently washed with DI water and freeze-dried to record the dry mass (*m*_*dry*_) left after degradation. The mass loss of the PLGA micronetworks was calculated as

1 $$mass\;loss\;\left(\%\right)=\frac{m_{ini}-m_{dry}}{m_{ini}}\times 100$$

### pH Measurements

pH was measured with a glass-bodied combination pH electrode (InoLab pH 730, WTW GmbH, Weilheim, Germany). The pH meter was calibrated with three buffer solutions: pH 4.01, pH 7.00, and pH 10.01. After recovering the PLGA micronetworks from the jars at predetermined time points, the pH value of the degradation media was recorded.

### Gel Permeation Chromatography

Gel permeation chromatography (GPC) was employed to obtain weight-average molecular weights ( $\overset{®}{M_{w}\;}$ ). μMESH samples incubated for 0, 7, 14, and 21 days in either buffered or unbuffered aqueous media were recovered and processed. At longer incubation times, molecular weight values decreased below the resolution limits of the GPC system, precluding reliable quantification. Prior to analysis, samples were freeze-dried, then dissolved in *N*,*N*-dimethylformamide (DMF) with 0.1% LiBr at 50 °C to a concentration of 10 mg/mL for 8 h and finally filtered through a 0.22 μm PTFE filter. GPC was performed using an integrated OMNISEC system (Malvern Panalytical Ltd., UK) equipped with a D6000 M and a D2500 column (10 and 6 μm particle size, both 300 × 8 mm) and a triple detection method (refractive index, a viscometer, and a dual angle light scattering detector at 15° and 90°). DMF with 0.1% LiBr was used as the eluent at a temperature of 50 °C and a flow rate of 1 mL/min. The system was calibrated using poly­(methyl methacrylate) (PolyCal standards, Malvern Panalytical Ltd., UK) 51 kDa narrow standard with known dispersity, intrinsic viscosity, and d*n*/d*c*. For each condition, measurements were averaged, and the $\overset{®}{M_{w}\;}$ values were plotted against time.

### Scanning Electron Microscopy Characterizations

The geometrical attributes of the different μMESH configurations were examined by electron microscopy. Specifically, samples were sputter-coated with 10 nm of gold and then imaged at 10 keV using an analytical low-vacuum SEM observation (SEM–JSM-6490, JEOL Ltd., Tokyo, Japan). Furthermore, electron microscopy was used to monitor topographical and morphological changes over time. For each configuration, 5 × 5 mm square μMESH were placed into 35 × 10 mm Petri dishes filled with 3 mL of DI water or 0.1 M PBS pH 7.4 and incubated at 37.0 ± 0.1 °C. At predetermined time points, namely 7, 14, 30, and 60 days, μMESH was removed from the medium, carefully rinsed with 3 mL of DI water, and dried at RT.

### Drug Loading and Release

To fabricate μMESH containing the model drug docetaxel (DTX), known amounts of the drug were added to the original PLGA solution before filling the ridges between the PVA square micropillars. For this experiment, chloroform (CHCl_3_) was used as the organic solvent to prevent drug precipitation due to its lower solubility in ACN. The loading of DTX into the PLGA micronetwork and its release over time were quantified by HPLC (1260 Infinity, Agilent Technologies, U.S.A.), using UV detection at 230 nm, based on external calibration curves. Drug loading efficiency was determined by dissolving DTX-loaded μMESH (*n* ≥ 5) in a solution of acetonitrile and water with a 1:1 ratio, followed by sonication and centrifugation at 18,000 *g* for 5 min. Entrapment efficiency (EE) was defined as the percentage of the initially added drug that was successfully incorporated into a full-size μMESH, according to the following equation

5 $$EE=\frac{drug-loaded\;per\;\mu MESH\times n_{\mu MESH}}{drug\;input\;into\;a\;full-sized\;\mu MESH}\times 100$$

where *n*_μMESH_ is the number of smaller, 5 × 5 mm μMESH samples obtained from a full-size μMESH.

To estimate the cumulative amount of released drug, one DTX-loaded μMESH was placed in a small tube (*n* ≥ 5) with 0.2 mL of physiological solution and incubated at 37.0 ± 0.1 °C. This relatively small volume was used to mimic the conditions that μMESH would encounter *in vivo* following intracranial implantation. Note that a volume of 0.2 mL corresponds to 200 mm^3^ or a tumor with a mass of approximately 0.2 g. At each time point (1, 3, and 6 hours and 1, 3, 7, 14, 21, 28, 42, 49, and 60 days), the samples underwent centrifugation at 18,000 *g* for 5 min, the supernatants were collected, and the tubes were replenished with fresh PBS. The amount of DTX released into the solution was cumulatively determined, mixing the supernatant with acetonitrile at a 1:1 ratio and performing HPLC analysis. At the end point, the amount of drug remaining inside the devices was determined by dissolving the residual PLGA micronetworks.

### Qualitative Characterization in Healthy Mice

The in vivo degradation study was conducted using 6 week-old male C57BL/6J mice (Charles River, Calco, Italy). All procedures were conducted in compliance with the principles of the “3Rs” (Replacement, Reduction, and Refinement) to minimize animal suffering and the number of animals used. Surgical procedures were performed under stereotaxic guidance to ensure precise device implantation. Animals were anesthetized with isoflurane (4% for induction and 1.5–2% for maintenance), and body temperature was maintained using a homeothermic monitoring system (RWD ThermoStar, RWD Life Science Co., Ltd., Shenzhen, China). The skin was disinfected sequentially with povidone-iodine solution (Meda Pharma S.p.A.) and 70% ethanol. After local administration of lidocaine, a midline scalp incision was made to expose the cranium. For each hemisphere, an implantation site was identified at anteroposterior (AP)–1.5 mm from the bregma and mediolateral (ML) ± 1.4 mm from the midline. At each site, a circular bone window (2.7 mm in diameter) was created using a trephine drill to expose the cortical surface after careful removal of the meningeal membrane. The 20 × 20 μMESH and FLAT devices were then placed on the cortical surface of the right and left hemispheres, respectively. All implants were sterilized by ultraviolet germicidal irradiation (UVGI) prior to implantation. After device placement, the bone fragments were repositioned, and the surgical wounds were closed with sutures. At the end of the surgical procedure, animals received intramuscular ketorolac for analgesia and were kept under a heating lamp until full recovery from anesthesia. Mice were housed in ventilated cages with ad libitum access to food and water under controlled environmental conditions (temperature: 21 ± 2 °C; relative humidity: 50 ± 10%; light/dark cycle: 12/12 h). At predetermined time points (3, 7, 14, 21, 28, 42, and 56 days postimplantation), animal body weight and general behavior were monitored, and images of the surgical site were collected. At the final experimental end point, clinical parameters including oxygen saturation, heart rate, and respiratory rate were recorded using a MouseOx Plus Pulse Oximeter (STARR Life Sciences Corp., Oakmont, PA, USA). Animals were then euthanized, and brain tissues were collected and processed for histological evaluation.

### Histological Analyses

Brain samples were collected at the experimental end points (1-, 3-, and 8 weeks postimplantation). To minimize mechanical damage during brain isolation and skull removal, the entire brain, together with the surrounding cranial bones, was harvested and immediately immersed in 4% paraformaldehyde in PBS (Santa Cruz Biotechnology Inc., Dallas, USA) for 1 week at 4 °C. Following fixation, samples were washed three times in PBS and transferred to a decalcifying solution (10% EDTA, pH 7.5–8.0, in 1× DPBS) to soften the bone matrix and allow sectioning. After decalcification, tissues underwent cryoprotection by sequential incubation in 20% and 30% sucrose solutions in PBS, each for at least 24 h at 4 °C or until complete sinking. Then, tissues were frozen in liquid nitrogen vapors and stored at −80 °C until further processing. Prior to sectioning, specimens were embedded in Surgipath FSC 22 Frozen Section Compound (Leica Biosystems, Deer Park, USA). Serial 20 μm-thick coronal sections were prepared to achieve whole-brain coverage on each slide (the detailed procedure is described in Spanò et al.^17^). Slides were stored at −20 °C until staining.

To evaluate implant resorption and tissue remodeling, hematoxylin and eosin (H&E) staining was performed. Hematoxylin solution, Gill No. 2 (Sigma-Aldrich GHS232, Merck KGaA, Darmstadt, Germany) and Eosin Y Solution, Alcoholic (Sigma-Aldrich HT110132, Merck KGaA, Darmstadt, Germany) were prepared according to the manufacturer’s instructions. Briefly, sections were incubated in Hematoxylin for 7 min and in Eosin for 10 s at room temperature, followed by dehydration in graded ethanol, clearing in xylene, and mounting with Fisher Chemical Permount Mounting Medium (Fisher Scientific SP15–500, Thermo Fisher Scientific, Waltham, USA). Images were acquired at 5× and 40× magnification using a Zeiss Axio Observer inverted microscope (Carl Zeiss Microscopy, Oberkochen, Germany).

### Statistical Analysis

Data are displayed as mean ± standard deviation (*n* ≥ 3). For multiple comparisons (i.e., experiments involving three or more groups), the Brown–Forsythe test was used to assess variance equality across groups, followed by a one-way analysis of variance (ANOVA). The two-sided log-rank test was applied to determine statistical significance. For experiments involving time-dependent measurements, data within the linear range were fitted by least-squares linear regression. Slopes obtained for each group were compared by nonlinear regression using a global fitting approach and an extra-sum-of-squares F-test to assess whether slope parameters differed significantly among groups. Statistical significance was set at *p* < 0.05. All statistical analyses and graphs were generated using GraphPad PRISM 9.5.1.

## Results

### Fabrication and Morphological Characterization of Micropatterned PLGA Films

μMESH is a dual-compartmentalized polymeric system consisting of a PVA microlayer integrated with a PLGA micronetwork (Figure 1A,B).^16,18^ μMESH enables the local, sustained release of various therapeutic agents over periods ranging from several days to months, making it particularly suited for the treatment of high-grade tumors using potent drugs that, if administered systemically, would not accumulate within the malignant tissue at sufficient levels and cause intolerable side effects. The archetypal configuration of μMESH −20 × 20 μMESH with square openings of 20 μm – has demonstrated therapeutic efficacy in preclinical animal models of orthotopic glioblastoma.^16,18^ As detailed in the Methods section, μMESH is fabricated through a top-down approach, summarized in Figure 1A. The first fabrication step is crucial for accurately defining the microarchitecture of μMESH and its PLGA film, as it determines the size and shape of an array of micropillars on a master silicon template by adjusting the direct laser writing parameters. The designed micropattern is then transferred into a PVA microlayer via an intermediate PDMS template. The PVA microlayer replicates the exact geometrical features of the silicon master.

In this work, four specific μMESH configurations were considered, corresponding to PVA microlayers displaying regular arrays of square micropillars with edge lengths of 5, 10, 20, and 50 μm (Figure 1C–first row). Then, a PLGA solution was carefully applied over the PVA microlayers to uniformly fill the empty channels (ridges) between the square micropillars (Figure 1C–second row). Confocal fluorescent images of the PLGA microlayers, loaded with the green fluorescent molecule curcumin, are shown in Figure 1D. While the height of the PVA micropillars (i.e., the thickness *h* of the PLGA strands in Figure 1B) is kept constant, the distance between adjacent PVA micropillars (i.e., the width *w* of the PLGA strands in Figure 1B) was adjusted according to the edge length of the PVA micropillars (i.e., the size *a* of the square openings in the PLGA micronetwork, as in Figure 1B). The table in Figure 1E summarizes the geometrical properties of the μMESH configurations. Using the archetypal 20 × 20 μMESH as a reference and having fixed the thickness *h* of the PLGA strands at 5 μm, the width *w* of the PLGA strands increases to 7.5 μm for the 50 × 50 μMESH to ensure adequate mechanical strength and to 5 μm for the 10 × 10 and 5 × 5 μMESH to allow the reduction in size of the square openings. This results in μMESH configurations with varying surface areas, *S*_*a*_, and volumes, *V*, of the PLGA micronetwork. Specifically, the surface area *S*_*a*_ of one single 20 × 20 μMESH was ∼31.3 × 10^6^ μm^2^, increasing to ∼50.1 × 10^6^ μm^2^ for the 10 × 10 μMESH and ∼62.6 × 10^6^ μm^2^ for the 5 × 5 μMESH, while it decreased to ∼19.8 × 10^6^ for the 50 × 50 μMESH. The volume *V* filled with PLGA was ∼30.8 × 10^6^ μm^3^ and ∼32.6 × 10^6^ μm^3^ for the 20 × 20 and 50 × 50 μMESH, respectively, almost doubling in volume for the 10 × 10 μMESH, and nearly 3-fold for the 5 × 5 μMESH (Figure 1E). Consequently, the surface area-to-volume ratio (*S*_*a*_/*V*) changed too with the μMESH configurations, being ∼1.02 for the 20 × 20 μMESH, reduced to 0.72 and 0.67 for the 10 × 10 and 5 × 5 μMESH, and to 0.65 for the 50 × 50 μMESH.

In the following, the various μMESH configurations are compared to FLAT, which represents a conventional, nonmicropatterned PLGA–PVA bilayer, with no pillars and openings (*S*_*a*_ ≈ 50.1 × 10^6^ μm^2^, *V* ≈ 125 × 10^6^ μm^3^, and *S*_*a*_/*V* = 0.40).

The geometry of the micropatterned PLGA film is expected to influence its degradation process, as well as its pharmaceutical and biological performance. Previous studies have shown that PVA can influence the degradation rates of PLGA micro- and nanoparticles.^7,19^ For this reason, in the present work, the PVA microlayer in μMESH was designed to rapidly dissolve in aqueous solution and therefore not interfere with long-term degradation processes. Specifically, because PVA dissolution in water is promoted by lower degrees of hydrolysis and lower molecular weights,^10^ a ∼10 kDa PVA with a 80% degree of hydrolysis was used.

Next, the behavior of the four micropatterned PLGA films and the FLAT control was evaluated over time using three complementary analytical approaches. First, bulk erosion was quantified by monitoring mass loss and independently verified by ^1^H-qNMR analysis. Second, polymer-chain scission was assessed by measuring the evolution of the molecular weight distribution using gel permeation chromatography (GPC). Third, the drug release kinetics were evaluated by measuring the amount of docetaxel (DTX) released over time by high-performance liquid chromatography (HPLC). Together, these techniques provided a comprehensive characterization of the degradation kinetics and mechanisms across all geometries.

### Erosion of Micropatterned PLGA Films – Mass Loss by Weight

After placing a full μMESH in DI water for 2 h to dissolve the PVA microlayer, the remaining micropatterned PLGA film was swabbed with paper and dried at room temperature, resulting in a wrinkled, nontransparent film as shown in Figure 2A **(left)**. Then, hydrolytic erosion was assessed by measuring loss in weight over 60 days of incubation in PBS (pH 7.4) and DI water at 37 °C. Representative images of a partially degraded PLGA film at 7 days postincubation in DI water and PBS are also shown in Figure 2A **(right)**. The pH variation of both incubation media over time is shown in Figure 2B, whereas PLGA mass variations over time are reported in Figure 2C,D for PBS and DI water, respectively. To ensure comparability across geometries, the number of μMESH units per experiment was adjusted to yield the same initial mass of PLGA (15 ± 0.05 mg) for all tested configurations. Specimens were incubated without stirring, as body fluids move slowly through solid tissues, which represent the typical implantation sites for drug delivery systems like μMESH.

**Figure 2:**
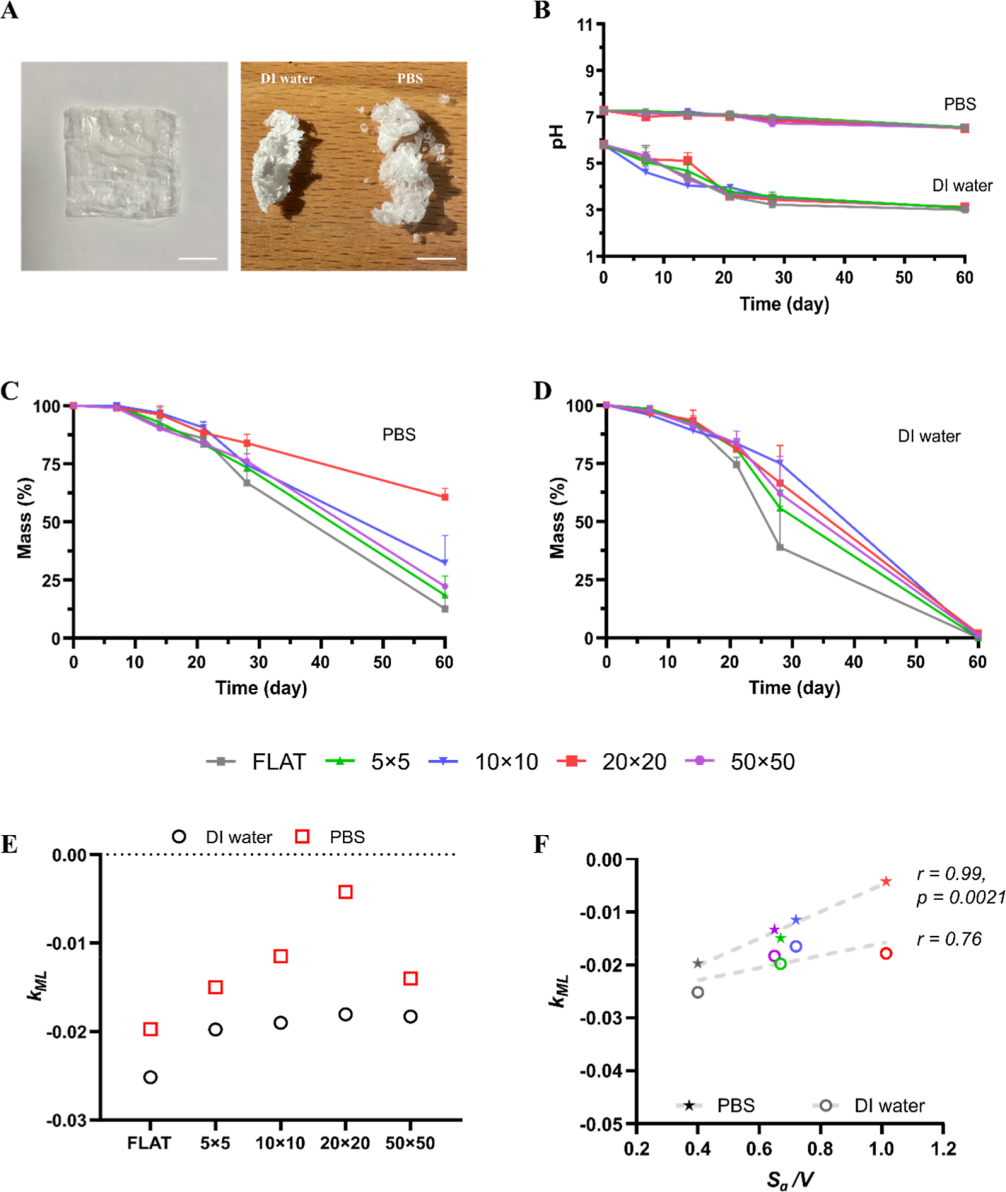
Degradation of micropatterned PLGA films – mass loss by weight. (A) Image of a micropatterned PLGA film in an aqueous solution (no PVA microlayer) (***left***). Images of PLGA films incubated for 7 days in DI water and PBS (***right***). (Scale bars: 1 cm). (B). Change in pH versus time for all tested micropatterned PLGA films and FLAT, in DI water and PBS. (C–D) Mass-loss vs time for the micropatterned PLGA films and FLAT in PBS and DI water, respectively. (E) Rate of mass loss *k*_*ML*_ for the micropatterned PLGA films and FLAT in DI water and PBS. Note that the rate is negative due to mass loss. (F) Scatter plots of *k*_ML_ vs *S*_*a*_/*V* for all tested micropatterned PLGA film configurations and FLAT, in PBS (stars) and DI water (open circles). Pearson’s correlation coefficient (r) and the relative statistical significance (p-value), shown on the graph adjacent to the regression line, were computed to quantify the linear association between variables.

PLGA hydrolysis is well-known to induce acidification of the surrounding medium as byproducts diffuse out of the matrix. This was evident in DI water, where the pH progressively decreased from approximately 5.9 ± 0.10 at the start of incubation to 3.0 ± 0.10 on day 60 for all μMESH configurations and FLAT, with only minor variations among geometries during the first 3 weeks (Figure 2B). In contrast, no substantial pH change was observed in PBS, where the pH declined slightly from approximately 7.2 ± 0.10 on day 0 to 6.5 ± 0.10 on day 60. Similar results were obtained in an independent assay in which μMESH and FLAT samples were allowed to degrade within Float-A-Lyzer dialysis devices under sink-like conditions, without removal of the PVA microlayer, to assess any contribution to medium acidification and the overall degradation process (Figure S1). Despite differences in experimental configuration, the pH profiles of the tested platforms were comparable across both systems (Figure 2B and Figure S1), indicating that neither the setup nor the presence of the PVA microlayer significantly affected medium acidification over time.

In DI water, as greater acidification of the medium occurred, a higher mass loss was expected. Mass loss remained below 10% for all tested samples during the first 7 days of incubation (Figure 2D). From day 7 onward, degradation progressed more rapidly, with a significant variation observed by day 28 among the various micropatterned PLGA films and FLAT. The 10 × 10 and 20 × 20 films had the lowest mass loss, corresponding to approximately 40%, whereas FLAT showed the fastest erosion, reaching approximately 60%. By the 60 day end point, all tested samples had been fully degraded, with no residual mass remaining. Physical examination revealed that the micropatterned PLGA films softened immediately upon immersion in DI water, suggesting rapid water diffusion into the PLGA strands. With continued immersion, the film became increasingly cloudy and whitish, indicative of changes in refractive index associated with water-rich domains. After drying, all specimens shrank in size, and their brittleness increased with longer incubation times (Figure 2A, ***right***). At later time points, the dried PLGA residues appeared as a compact, viscous mass.

In PBS, mass loss was again nearly zero during the first 7 days and then increased progressively over the following 7 weeks, with a noticeable acceleration after approximately 3 weeks (Figure 2C). Among all micropatterned PLGA films, the 20 × 20 μMESH exhibited the lowest erosion rate, with mass loss of roughly 10% at 21 days and only 40% at 60 days. In contrast, the 10 × 10 μMESH showed approximately 70% mass loss on day 60, while the 50 × 50 and 5 × 5 μMESH degraded by about 80%. Even in a physiological buffer, FLAT displayed the fastest erosion, with nearly 90% mass loss by day 60. In striking contrast to the DI water condition, residual PLGA film material was still present at the end of the experiment in PBS. PBS is commonly used as a standard medium in degradation and drug-release studies because it maintains pH and osmolarity comparable to those of body fluids.^20^ However, to more closely mimic in vivo conditions, a 5 × 5 micropatterned PLGA film was incubated in artificial cerebrospinal fluid, showing mass-loss and pH trends comparable to those observed in PBS (Figure S2).

Additionally, similar trends were observed when quantifying the remaining PLGA mass of μMESH platforms (5 × 5 mm, different starting polymeric amount) incubated in either DI water or PBS using qNMR. The PULCON method was first validated (Figure S3), then applied for PLGA mass quantification (Figure S4), and subsequently used to assess erosion profiles (Figure S5).

Focusing on the erosion profiles, in PBS, the micropatterned PLGA films exhibited an initial lag phase during which mass loss was negligible for all microarchitectures, followed by a decay phase that was described using a pseudo-first-order kinetic model, consistent with previously reported results.^21^ Figure S6A shows the data plotted on a semilogarithmic scale, enabling clear identification of these two regimes. From day 21 to day 60, samples incubated in PBS displayed a quasi-linear decrease in mass across all configurations. Following the method described by Kenley et al.,^22^ data from day 21 onward were fitted using least-squares regressions to determine the rate of mass loss, *k*_*ML*_ (Figure S6B). Higher absolute values of *k*_*ML*_ indicate a faster erosion process. In PBS, *k*_*ML*_ values showed a strong dependence on microgeometry: correlation analysis revealed a high Pearson coefficient (r = 0.99, p = 0.0021), and the extra-sum-of-squares F-test confirmed statistically significant differences among configurations (*p* < 0.0001), supporting a clear relationship between erosion kinetics and the *S*_*a*_/*V* ratio (Figure 2F).

A similar analysis was performed for samples eroded in DI water, too. The semilogarithmic mass-loss profiles (Figure S6C) show a faster onset of the decay phase compared to PBS. The fitting of the decay region (Figure S6D) confirmed higher absolute *k*_*ML*_ values for all geometries, reflecting enhanced acidification and hydrolysis in the unbuffered medium, as summarized in the table in Figure S6E. In DI water, the micropatterned films exhibited comparable *k*_*ML*_ values, whereas FLAT showed a faster erosion rate. Despite this difference, correlation analysis between *k*_ML_ and *S*_*a*_/*V* yielded a Pearson coefficient of r = 0.76 with a nonsignificant p-value (∼0.14), and the extra-sum-of-squares F-test did not reveal statistically significant differences among configurations (p = 0.615), indicating that data obtained in DI water do not support a statistically meaningful dependence of erosion kinetics on microgeometry. This behavior is consistent with the absence of buffering capacity, which promotes rapid acidification and autocatalytic degradation, thereby masking geometry-dependent effects.

### Degradation of Micropatterned PLGA Films – Change in Molecular Weight

In addition to assessing mass loss by weight, the degradation of the micropatterned PLGA films was evaluated by monitoring changes in their molecular weight over time using gel permeation chromatography. Note that both the number-average ( $\overset{®}{M_{n}\;}$ ) and the weight-average ( $\overset{®}{M_{w}\;}$ ) molecular weights can be used to quantify polymer degradation. However, $\overset{®}{M_{w}\;}$ reflects more reliably the progression of chain scission that precedes and ultimately drives mass loss, and correlates more closely with the mechanical properties.^11^ For these reasons, $\overset{®}{M_{w}\;}$ was selected to follow degradation at a macromolecular level (Figure 3A,B). Representative GPC chromatograms illustrating $\overset{®}{M_{w}\;}$ shifts over time are shown in Figure S7. The data revealed an apparent first-order degradation behavior, as confirmed by the corresponding semilogarithmic plots in Figure S8A,B, with fitting parameters summarized in the table in Figure S8C. The degradation rate constant (*k*_DEG_) was calculated from the slope of the least-squares regression fit for each configuration and is reported in Figure 3C. Overall, no substantial differences in degradation rates were observed between incubation media for most geometries. An exception was the 20 × 20 micropatterned PLGA film, which degraded more slowly in PBS than in DI water. Although this difference was not statistically significant (*p* > 0.05), the 20 × 20 configuration exhibited the lowest absolute *k*_DEG_ value in PBS, indicating the slowest molecular weight decay under buffered conditions.

**Figure 3:**
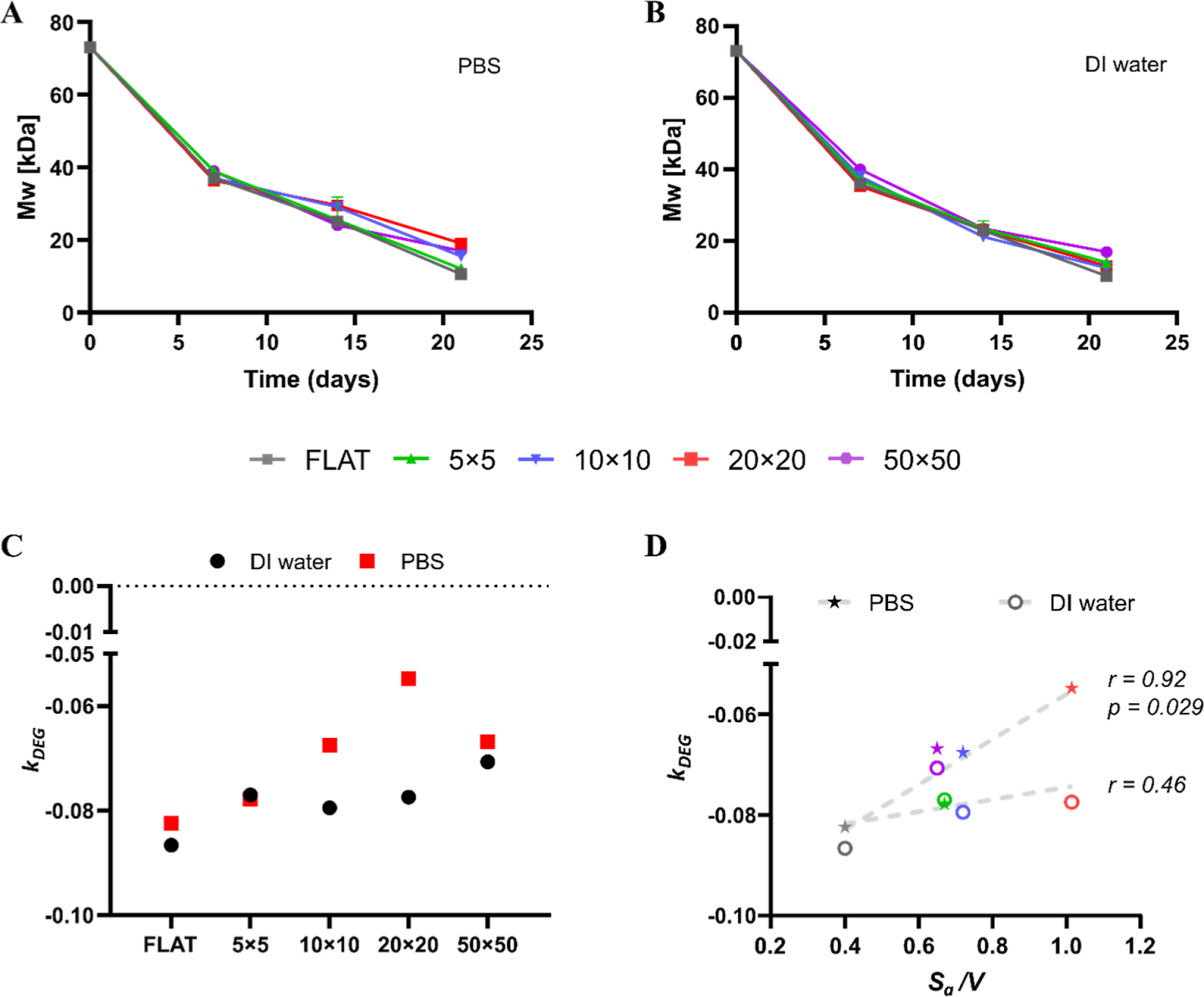
Degradation of micropatterned PLGA films – change in molecular weight. (A–B) Evolution of PLGA weight-average molecular weight ( $\overset{®}{M_{w}\;}$ ) as a function of time for micropatterned PLGA films and FLAT in PBS and DI water, respectively. (C) Rate of degradation (*k*_DEG_) for the micropatterned PLGA films and FLAT in DI water and PBS. Note that the rate is negative because of the $\overset{®}{M_{w}\;}$ decrease over time. (D). Scatter plots of *k*_DEG_ versus *S*_*a*_/*V* for all tested micropatterned PLGA film configurations and FLAT, in PBS (stars) and DI water (open circles). Pearson’s correlation coefficient (r) and the relative statistical significance (p-value), shown on the graph adjacent to the regression line, were computed to quantify the linear association between variables.

Consistent with the erosion analysis, degradation rates in PBS displayed a geometry-dependent trend. Specifically, higher *S*_*a*_/*V* were associated with lower absolute *k*_*DEG*_ values and thus slower $\overset{®}{M_{w}\;}$ decay (Figure 3D). Pearson’s correlation analysis confirmed a strong, statistically significant correlation in PBS (r = 0.9165, p = 0.0286). However, the extra-sum-of-squares F-test did not reveal statistically significant differences among configurations in PBS (p = 0.2735), indicating that the fitted slopes were not significantly different when directly compared.

In DI water, the correlation between *k*_*DEG*_ and *S*_*a*_/*V* was weaker and not statistically significant (r = 0.46, p = 0.2146). In agreement with this observation, the extra-sum-of-squares F-test yielded no significant differences among configurations (p = 0.8365), reflecting the increased variability in degradation behavior in the absence of buffering capacity.

To further evaluate the role of individual geometric parameters, additional correlation analyses were performed for filament width (*w*) and opening size (*a*). These analyses did not reveal statistically significant correlations for either *k*_ML_ (Figure S9A, B) or *k*_*DEG*_ (Figure S9C, D), and the corresponding Pearson coefficients and coefficients of determination (R^2^) are summarized in the table in Figure S9G. These results confirm *S*_*a*_/*V* as the dominant geometric parameter in buffered media, where diffusion-driven transport governs degradation behavior.

### Morphological Alteration of the Micropatterned PLGA Films

Changes in the morphology of the PLGA films and FLAT over time were evaluated in DI water and PBS using scanning electron microscopy (SEM), as summarized in Figure 4. Surface roughening and the formation of pores, holes, and cracks were used as key indicators of the degradation process. In DI water, PLGA samples began to exhibit these degradation features as early as 14 days, with both their size and abundance increasing progressively over time. After 30 days of incubation, all film configurations had lost their original geometry, as the PLGA strands collapsed and bundled together, forming a porous and amorphous structure. By 60 days, the structures had collapsed and shrunk further with pores and holes closing, yielding a continuous, irregular solid layer.

**Figure 4:**
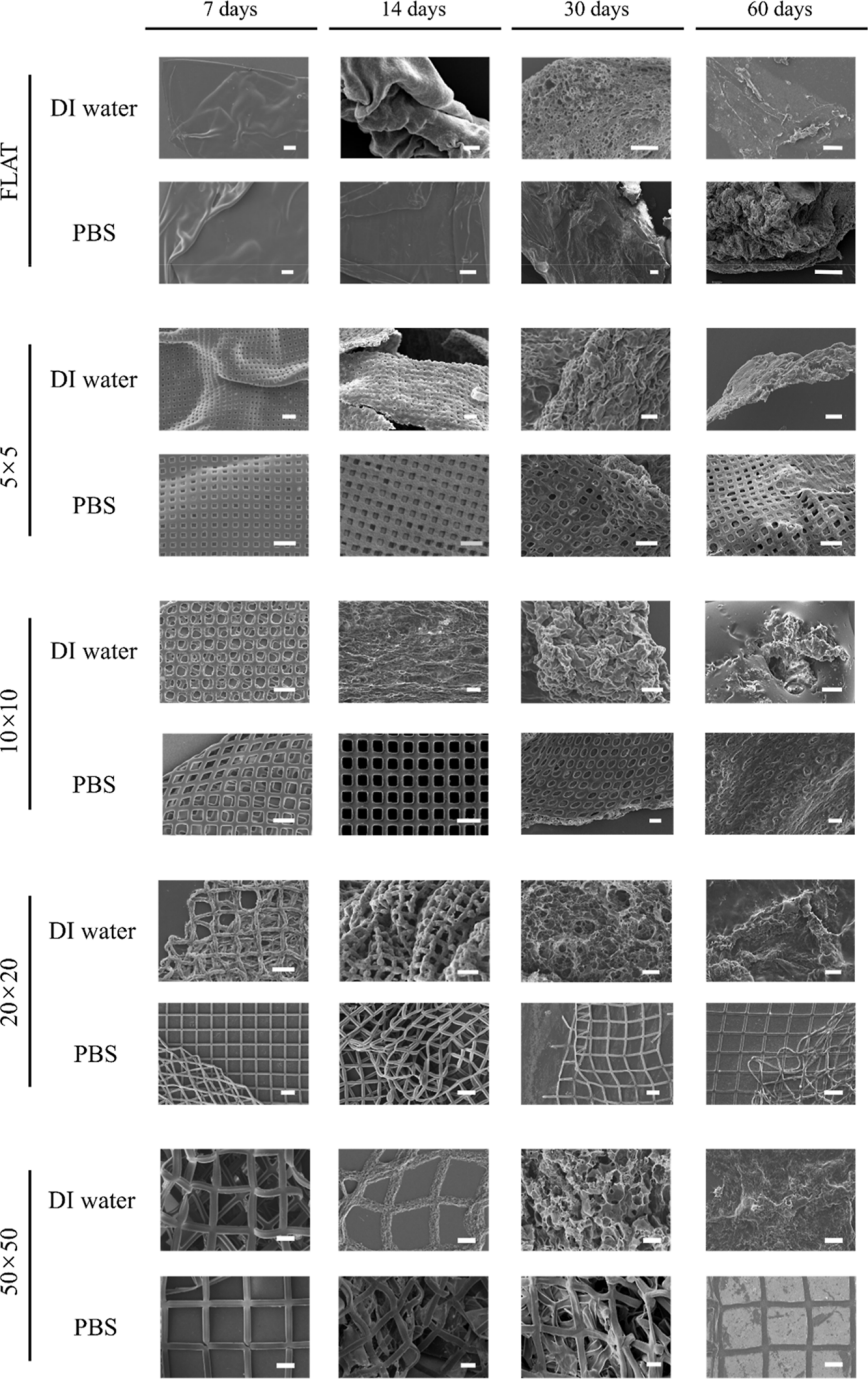
Morphological alteration of the micropatterned PLGA films over time. Scanning electron microscopy images showing morphological changes in all tested micropatterned PLGA films and FLAT after 7, 14, 30, and 60 days of incubation in DI water and PBS. From left to right: FLAT (control), 5 × 5, 10 × 10, 20 × 20, and 50 × 50 micropatterned PLGA films. Scale bars: 100 μm for FLAT and 20 μm for all other configurations.

In contrast, micropatterned PLGA films incubated in PBS retained their geometric features for a longer period. However, signs of heterogeneous degradation appeared in the 5 × 5 and 10 × 10 films after 14 days, as indicated by the widening of openings, progressive distortion of their originally square geometry, and an increased presence of pores within the bulk. By comparison, the 20 × 20 and 50 × 50 films preserved their distinct architecture for up to 60 days, although gradual thinning of the PLGA strands was evident over time (Figure S10A). Differently, the FLAT configuration progressively lost its planar geometry during incubation, undergoing structural rearrangement and collapse into a dense, aggregated morphology, with no preservation of the initial planar structure (Figure S10B).

### Drug Loading and Release from the Micropatterned PLGA Films

The ability of μMESH to efficiently load and deliver therapeutic agents, including docetaxel (DTX), has been previously demonstrated.^16,18^ Because of DTX hydrophobicity, the drug was codissolved with PLGA in chloroform (CHCl_3_), and the resulting viscous solution was deposited into the free spaces between the PVA micropillars (ridges). Since μMESH was originally fabricated using ACN, a comparative analysis between ACN- and CHCl_3_-based formulations was first performed to assess potential effects of the solvent on morphology, geometric features, and pharmacological behavior. No significant differences were observed: SEM images confirmed comparable microstructures (Figure S11A,B), while similar drug entrapment efficiencies and release profiles were obtained (Figure S11C,D), indicating that both morphology and performance were preserved. In this study, a full-size 20 × 20 μMESH was loaded with 1 mg of DTX, yielding individual 20 × 20 micropatterned PLGA films containing 15.49 ± 2.09 μg of drug and an entrapment efficiency (EE) of 56 ± 7.34%. Note that a full-size μMESH layer has an edge length of 30 mm, whereas each micropatterned PLGA film is 36 times smaller. To ensure comparability among geometries, the amount of drug incorporated into each PLGA film was adjusted to maintain a constant PLGA-to-DTX mass ratio. As a result, FLAT exhibited the highest drug loading (98.85 ± 21.67 μg), followed by the 5 × 5 (80.18 ± 6.51 μg), 10 × 10 (49.20 ± 5.96 μg), and 50 × 50 (19.13 ± 5.62 μg) micropatterned PLGA film. The measured drug loading amounts and encapsulation efficiencies for all tested configurations are presented in Figure 5A and B, respectively. Higher EE values were observed for FLAT (67.35 ± 13.79%), 5 × 5 (67.35 ± 17.28%), and 10 × 10 (73.01 ± 3.53%) micropatterned films, likely due to their surface geometries, which either lack openings (FLAT) or contain smaller ones (5 × 5 and 10 × 10), thereby reducing the loss of drug during fabrication. Drug loading amounts and EE values were calculated by analyzing multiple units of micropatterned PLGA films collected from various locations across a full-size μMESH (30 × 30 mm).

**Figure 5:**
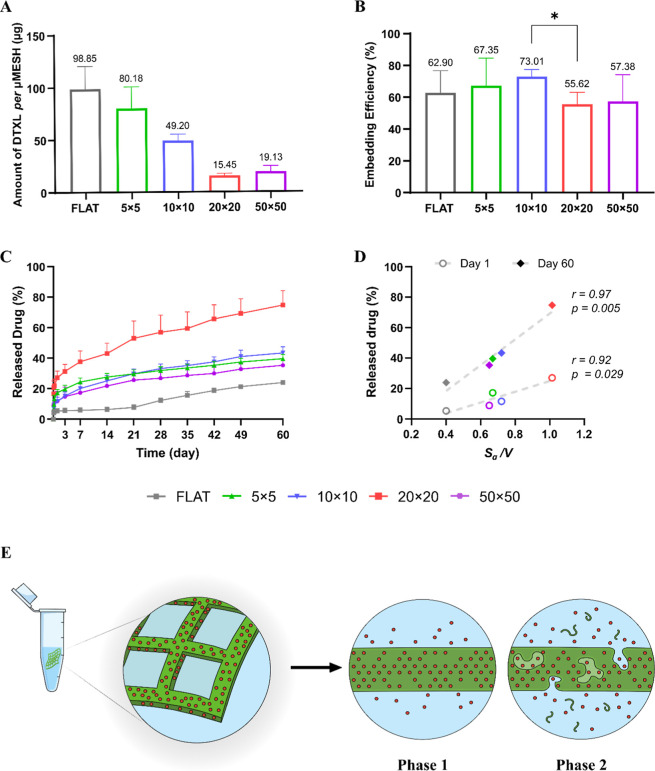
Pharmacological characterizations of micropatterned PLGA films. (A) Amount of docetaxel (DTX) loaded into micropatterned PLGA films with different architectures and FLAT. (B) Entrapment efficiency (EE) for all the tested micropatterned PLGA films with different architectures and FLAT. (C) Cumulative release of DTX from all tested micropatterned PLGA films and FLAT over a 60 day period. (D) Scatter plots of cumulative DTX released (%) on day 1 (open circles) and 60 (diamonds) versus *S*_*a*_/*V* for all tested micropatterned PLGA film configurations and FLAT. Pearson’s correlation coefficient (r), shown on the graph adjacent to the regression line, was computed to quantify the linear association between variables. (E) Schematic representation of the experimental setup and the proposed release mechanisms: *Phase I*, characterized by rapid diffusion from the films surface, followed by *Phase II*, in which drug transport occurs through the polymer matrix and the micropores generated by polymer erosion, resulting in a more sustained release.

The pharmaceutical properties of the micropatterned PLGA films carrying DTX were further evaluated by characterizing drug release over time and examining potential correlations with the *S*_*a*_/*V* ratio and degradation behavior. Release profiles are shown in Figure 5C, while cumulative release values are reported in Figure S11E. Note that the release assay should be interpreted as a localized, finite-volume model that captures restricted diffusion around μMESH, rather than as a full recapitulation of in vivo release conditions. DTX release exhibited a biphasic profile. Phase I consisted of an initial, more rapid release, observed across all configurations, with the 20 × 20 film displaying a significantly higher release within the first 24 h than any other geometry. This behavior is associated with the rapid diffusion of the most superficially located drug molecules from the PLGA film characterized by the highest *S*_*a*_/*V* value. During Phase II, a slower sustained release was observed with 37.60 ± 7.07% of the DTX cargo being released from the 20 × 20 PLGA film on day 7, whereas FLAT released only 5.25 ± 1.52% despite its substantially higher initial drug loading. By the end of the observation period, the 20 × 20 micropatterned film had released nearly its entire payload (74.78 ± 9.45%), followed by the 10 × 10 (43.38 ± 4.04%), 5 × 5 (39.6 ± 2.74%), 50 × 50 (35.30 ± 3.08%), and FLAT (23.92 ± 0.77%).

The cumulative percentage of DTX released exhibits a positive correlation with *S*_*a*_/*V*. Configurations with higher *S*_*a*_/*V* values released a larger fraction of their drug payload at both 24 h (r = 0.92, Figure 5D, **lower line**) and day 60 (r = 0.97, Figure 5D, **upper line**). This trend indicates that increasing *S*_*a*_/*V* enhances drug release, likely by shortening diffusion pathways and increasing the interfacial area between the polymer matrix and the surrounding medium during the early diffusion dominated phase, and by facilitating more efficient erosion-mediated release at later stages. Consistent with the analysis performed for *k*_ML_ and *k*_*DEG*_, the possibility of correlating drug release rates with individual geometric parameters was investigated; however, no meaningful correlations were observed, consistent with the analysis reported in Figure S9E,F. In contrast, the strong linear correlations observed here, as quantified by Pearson’s correlation coefficients in Figure 5D, highlight *S*_*a*_/*V* as a key determinant of release kinetics in micropatterned PLGA films.

The biphasic release behavior and the underlying transport mechanisms are schematically illustrated in Figure 5E.

### Preliminary Qualitative Analysis of Micropatterned PLGA Films Interaction with Brain Tissue

To investigate how microarchitecture influences erosion kinetics in a physiological environment and to assess the interaction between PLGA films and cerebral tissue in vivo, experiments were performed in healthy mice following the experimental design of Figure 6A. A 3 × 3 mm cranial window was created, and PLGA films with an edge size of 2.5 mm were positioned at bregma coordinates AP – 1.5 mm. A single FLAT film was implanted at ML + 1.4 mm (left hemisphere), and a 20 × 20 micropatterned PLGA film was placed at ML – 1.4 mm (right hemisphere). The total polymer mass was kept constant across conditions to ensure that only geometrical features differed. To match the total polymer mass of one FLAT, four 20 × 20 micropatterned PLGA films were stacked in a staggered manner. Implants were applied directly onto the cortical surface following trephination; the overlying bone was then repositioned.

**Figure 6:**
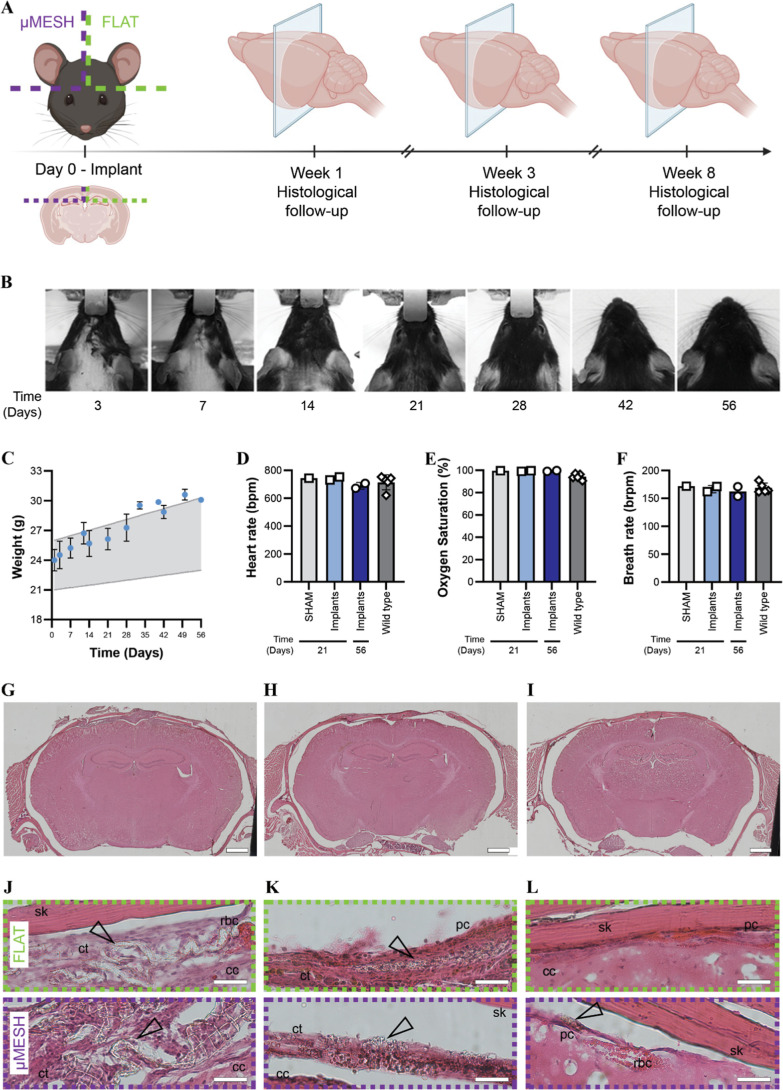
Histological analysis of μMESH interaction with brain tissue. (A) Schematic overview of the experimental workflow, highlighting key time points and study end points. Each animal received a μMESH implant in the right hemisphere and a FLAT implant in the left hemisphere. (B) Representative photographic images of the surgical site over time, demonstrating normal skin healing and hair regrowth. (C) Body-weight progression of implanted mice throughout the preclinical study. Growth rates were comparable to the normal growth curve of age-matched wild type animals (light gray area). (D–F) Clinical parameters - including heart rate (D), oxygen saturation (E), and respiratory rate (F) – measured at the final experimental end point. SHAM animals underwent surgery without implant placement. Orange dotted lines indicate average physiological values for healthy C57BL/6J mice under standard conditions (wild type). (G–I) Representative coronal brain sections collected at 1- (G), 3- (H), and 8 (I) weeks postimplantation (PI) (magnification: 5×; scale bar: 1000 μm). (J–L). High-magnification histological images of FLAT and μMESH implant sites collected at 1- (J), 3- (K), and 8 (L) weeks PI (magnification: 40×; scale bar: 50 μm). Both implants are positioned between the parietal bones (***sk***) and the underlying cerebral cortex (***cc***). At 1 week PI, connective-tissue (***ct***) fibers interlace with the transparent implant matrices (arrows), with inflammatory cells and red blood cells (***rbc***) present in the surrounding tissue. At 3 weeks PI, connective-tissue fibers remain visible and closely interact with the progressively degrading polymeric matrices (arrows). At 8 weeks PI, only a small μMESH fragment remains detectable (arrow), while no residual FLAT material is observed. Gold-stained phagocytic cells (***pc***) are present within both implant regions.

Throughout the study, no neurological or systemic alterations were observed for either the 20 × 20 micropatterned film or the FLAT. Animals exhibited normal wound healing and hair regrowth (Figure 6B), and normal weight gain (Figure 6C). All monitored clinical parameters, including heart rate, oxygen saturation, and respiratory rate, remained within physiological ranges and did not differ statistically from wild-type and SHAM animals (Figure 6D).

Macroscopic inspection of the brain and skull revealed no abnormalities (Figure S12). Histological sections collected at 1-, 3-, and 8 weeks post implantation (PI) showed no structural alterations in cerebral or surrounding bone tissue by H&E staining (Figure 6G–I). High-magnification imaging demonstrated progressive erosion of both geometries over time (Figure 6J–L). At 1 week PI, both FLAT and μMESH remained clearly visible, transparent, unstained by H&E, and encapsulated by a thin fibrous layer (Figure 6J). Notably, while the fibrous capsule was modest in both cases, it appeared slightly thicker around the FLAT implant, suggesting a higher degree of local inflammation for this configuration. The micropatterned PLGA film microarchitecture was still distinguishable at this stage, whereas the FLAT had lost its morphology and appeared as a swollen PLGA aggregate. Differences were more evident at 3 weeks PI: the FLAT had markedly decreased in size, while the micropatterned PLGA film appeared swollen but structurally preserved (Figure 6K). By 8 weeks PI, extensive erosion was evident for both geometries, with only isolated μMESH fragments remaining detectable (Figure 6L).

## Discussion

In bulk eroding polymers, mass loss is often characterized by an initial lag phase associated with the accumulation of acidic degradation byproducts, which lowers the internal pH, accelerates chain scission, and ultimately leads to matrix softening and collapse.^10,13,23^ Reducing diffusion length and increasing surface area, as achieved through micropatterning, can facilitate the outward diffusion of acidic species, mitigate autocatalysis, and promote more controlled erosion and degradation. To investigate these effects, four micropatterned PLGA films spanning a range of surface area-to-volume ratios, *S*_*a*_/*V*, from low (FLAT −0.40 μm^–1^) to intermediate (5 × 5, 10 × 10, and 50 × 50 μMESH −0.65 to 0.72 μm^–1^) and high (20 × 20 μMESH −1.02 μm^–1^), were fabricated and systematically investigated.

Consistent with previous reports,^3,13,24^ both degradation and erosion kinetics were strongly influenced by *S*_*a*_/*V*. Low *S*_*a*_/*V* configurations hindered water access and favored the retention of acidic oligomers, whereas higher *S*_*a*_/*V* promoted diffusive transport and reduced autocatalytic degradation. Accordingly, configurations with a higher *S*_*a*_/*V* ratio exhibited slower mass loss and reduced molecular weight decay. To ensure that these differences were only geometry-driven, the initial PLGA mass was kept constant across all tested configurations. Incubation in DI water was used to highlight the critical role of the surrounding medium in controlling hydrolysis, with PBS and aCSF better mimicking physiological conditions through buffering capacity. Degradation proceeded rapidly in DI water, where the pH dropped to approximatively 3 by day 60. In contrast, buffered media (PBS, aCSF), limited acidification and stabilized degradation kinetics. In PBS, erosion exhibited a geometry-dependent induction phase: low *S*_*a*_/*V* films began losing mass earlier, whereas higher *S*_*a*_/*V* configurations eroded more gradually. The erosion rate constants (*k*_*ML*_) correlated with *S*_*a*_/*V*, and molecular weight analysis indicated that geometry modulated the extent of autocatalysis rather than the overall bulk erosion mechanism. These observations can be rationalized within a diffusion-controlled framework, consistent with Fick’s laws of mass transport. The transport of degradation products is governed by the balance between their volumetric generation within the polymer matrix and their diffusion-driven removal at the polymer–aqueous interface, a relationship that scales with *S*_*a*_/*V* and determines the efficiency of mass transport. Diffusion occurs in all spatial directions, and the characteristic diffusion length (*L*_*D*_) depends on the geometry of the structure and can be approximated by the ratio between volume and surface area (*L*_*D*_ ∼ *V*/*S*_*a*_). As a result, configurations with higher *S*_*a*_/*V* exhibit shorter effective diffusion pathways, *L*_*D*_, enabling faster removal of low-molecular-weight degradation products and limiting their accumulation within the matrix. In contrast, individual geometric parameters, such as the opening size (*a*), do not directly influence the characteristic diffusion distance nor the bulk generation of acidic species, which is inherently volume-dependent.

Notably, the 20 × 20 μMESH film configuration displayed the slowest molecular weight decay, consistent with the shorter diffusion distance, which enables a faster escape of low molecular weight products to the aqueous phase. These species have the highest acidification capacity, so their loss is expected to reduce the rate of acid-catalyzed hydrolysis. Conversely, in DI water, the absence of buffering promotes rapid acidification and autocatalytic degradation, which minimizes geometry-dependent effects by reducing the contribution of diffusion-driven removal of acidic degradation byproducts. SEM analysis further revealed gradual thinning of the polymer filaments and expansion of the square without significant architectural collapse over 60 days in PBS. In contrast, FLAT, with its low *S*_*a*_/*V* ratio and the highest mass of PLGA (785.7 μg), eroded most rapidly, developing cracks and pores, followed by structure collapse into an aggregated, unstructured mass, which are clear signs of bulk erosion.^13,23^

All film configurations were loaded with DTX at a constant polymer-to-drug ratio to achieve a uniform drug distribution of approximately 2 wt % relative to the polymer across all configurations. All films exhibited biphasic release profiles, consisting of an initial burst phase (Phase I) followed by a sustained release phase (Phase II). The burst release was primarily driven by outward diffusion of drug molecules located at or near the micronetwork surface, with its magnitude scaling with the *S*_*a*_/*V* ratio – the larger the surface exposed per unit volume, the larger the outward drug flux, consistent with Fick’s first law. Accordingly, the 20 × 20 μMESH film exhibited the highest burst release (∼20%), followed by the 10 × 10, 5 × 5, and 50 × 50 μMESH films, while FLAT showed the lowest burst release (∼5%). During the sustained release phase, μMESH platforms largely preserved their overall geometry and underwent a progressive exfoliation process, characterized by a reduction in PLGA filament thickness without significant changes in microarchitecture, as evidenced by scanning electron microscopy. This gradual thinning led to a progressive increase in the *S*_*a*_/*V* ratio, thereby sustaining faster drug release. As a result, cumulative release increased with *S*_*a*_/*V*, with the 20 × 20 μMESH film releasing the largest fraction of its payload (∼75% at 60 days). In contrast, FLAT underwent substantial geometric and morphological rearrangements during degradation, including matrix folding and collapse, as documented by scanning electron microscopy. These changes generated a compact and highly tortuous structure that significantly increased the effective diffusion path length and restricted drug transport. This effect was further amplified by the physicochemical properties of DTX. As a hydrophobic compound (logP ∼ 3), DTX limits water uptake into the PLGA matrix and promotes competitive mass transport with low-molecular-weight acidic oligomers generated during degradation.^15,25^ The combined effects of increased tortuosity and competitive diffusion slow drug release despite extensive polymer mass loss, largely attributed to autocatalytic degradation within the film core. In addition, the confined release volume used in the assay (0.2 mL) likely imposed finite-sink conditions, further limiting DTX release, particularly from FLAT, which has the highest absolute drug loading. Together, these factors provide a consistent mechanistic explanation for why the FLAT configuration exhibited ∼80% mass loss at 60 days while releasing only ∼20% of the loaded DTX.

Finally, the 20 × 20 μMESH film and FLAT, sitting at the two extremes of the *S*_*a*_/*V* spectrum, were selected for a qualitative in vivo evaluation. At two months postintracranial deposition of both PLGA films, residual fragments were still detectable for the 20 × 20 μMESH configuration, whereas the FLAT had largely degraded. Erosion appeared moderately accelerated in vivo, potentially due to the presence of biological plasticizers and reactive species.^13,26^ Tissue responses were mild and consistent with the established biocompatibility of PLGA, with no macroscopic or histological abnormalities observed, in agreement with previous reports on implanted polymeric biomaterials.^27^ As both implants contained equivalent PLGA mass, differences in degradation and tissue integration are likely attributable to mechanical and interfacial effects, with the continuous FLAT geometry behaving as a stiffer, less compliant structure. Future studies will focus on immunohistochemical characterization of neural and immune responses to further elucidate the impact of implant micropatterning on local tissue remodeling.

## Conclusion

Employing the μMESH platform, which readily allows modulation of the surface area-to-volume ratio (*S*_*a*_/*V*) of PLGA films, we investigated the effects of micropatterning on degradation and erosion kinetics and drug release behavior. Higher *S*_*a*_/*V* ratios were associated with reduced mass loss, slower molecular-weight decay, and sustained release rates, predominantly driven by diffusion. In particular, the 20 × 20 μMESH film (*S*_*a*_/*V* = 1.02 μm^–1^) exhibited the highest structural stability, accompanied by sustained drug release over 60 days, highlighting the interplay among micropatterning, diffusion length, and degradation dynamics. In contrast, the FLAT film (*S*_*a*_/*V* = 0.4 μm^–1^) showed early signs of rapid degradation, with the formation of a compact mass that impaired drug release. In vivo, these trends were qualitatively preserved, with the 20 × 20 μMESH degrading more slowly than the unpatterned FLAT film. In both cases, the films exhibited excellent biocompatibility, even within sensitive cortical brain tissue. Overall, these findings indicate that the degradation behavior and permanence of PLGA films can be effectively tuned by tailoring their geometry alone, without altering the material composition. This strategy provides a robust framework for the rational design of implantable drug delivery films and may be broadly applicable to biomedical applications that require precise spatial and temporal control of therapeutic delivery.

## Supplementary Material



## Notes

The Supporting Information provides detailed experimental methods, additional in vitro and in vivo data, and supplementary analyses supporting the characterization of μMESH, including its degradation behavior, drug release, and biological response (PDF)

## ¶

Ludwig Boltzmann Institute for Nanovesicular Precision Medicine, Paris Lodron University of Salzburg, 5020 Salzburg, Austria
