# Supplementary material for: Programming Degradation and Drug Release Through Micropatterning of PLGA Films
Source: ACS Appl Mater Interfaces. 2026 May 13;18(20):28464–78. doi: 10.1021/acsami.6c04044 (PMC13220224; doi:10.1021/acsami.6c04044)
Supplement: Supplementary file 1 [file am6c04044_si_001.pdf]

SUPPORTING INFORMATION

PROGRAMMING DEGRADATION AND DRUG RELEASE THROUGH MICROPATTERNING OF PLGA FILMS

Irene Guerriero[1,2], Cristiano Pesce [1,3], Raffaele Spanò[1], Stefania Sganga[5], Nicola Tirelli[5], Anna Lisa Palange[1], Daniele Di Mascolo[1,4], Paolo Decuzzi[1,6] *

[1] Laboratory of Nanotechnology for Precision Medicine, Fondazione Istituto Italiano di Tecnologia, 16163, Genoa, Italy

[2] Department of Informatics, Bioengineering, Robotics and System Engineering, Università di Genova, 16145, Genoa, Italy

[3] Department of Pharmaceutical and Pharmacological Sciences, University of Padua, 35122, Padova, Italy

[4] Department of Electrical and Information Engineering, Politecnico di Bari, 70126, Bari, Italy

[5] Laboratory of Polymers and Biomaterials, Fondazione Istituto Italiano di Tecnologia, 16163, Genoa Italy

[6] Division of Oncology, Department of Medicine and Department of Pathology, Stanford University School of Medicine, Stanford, 94305 California, United States

CORRESPONDING AUTHOR. *Email: paolo.decuzzi@iit.it

SUPPORTING MATERIALS AND METHODS

Materials. Dimethyl sulfone ($DMSO_2$, TraceCERT, 99.99%), dimethyl sulfoxide anhydrous (DMSO), dimethyl sulfoxide-d6 99.9 atom % D (DMSO-d_6), sodium chloride (105 mM), potassium chloride (2.95 mM), magnesium chloride (4.62 mM), calcium chloride (2.34 mM), sodium carbonate (56.6 mM), disodium hydrogen phosphate dihydrate (0.337 mM), D-glucose (3.33 mM), L-ascorbic acid (1.14 mM), and bovine serum albumin (0.15 g) were purchased from Merck (Darmstadt, Germany). Disposable SampleJet-compatible NMR tubes with an outer diameter of 3 mm were used and purchased from Bruker Italia Srl (Milan, Italy). Float-A-Lyzer® G2 dialysis device (100 kDa cutoff) was purchased from Thermo Fisher Scientific (Segrate, Italy).

S1. Medium acidification during full μMESH degradation in a Float-A-Lyzer® G2 dialysis device. Dual-compartment μMESH were placed inside the Float-A-Lyzer® G2 dialysis device (100 kDa cutoff) filled with 2 mL of DI water or 0.1 M PBS (pH 7.4). The number of μMESH samples was adjusted to ensure that all devices contained the same initial amount of polymer (~ 0.5 mg), and the PVA microlayer was not removed prior to incubation. The dialysis devices were then inserted into 50 mL tubes containing 25 mL of the corresponding external medium (DI water or PBS). Tubes were placed in an incubator at 37.0 ± 0.1 °C under horizontal rotation at 70 rpm, and the pH of the outer medium was recorded at predetermined time points, namely 0, 7, 14, 28, and 60 days.

S2. Incubation in artificial cerebrospinal fluid. Erosion of micropatterned PLGA films was also evaluated in artificial cerebrospinal fluid (aCSF). To prepare 500 mL of aCSF, a beaker containing 500 mL of DI water was placed on a stir plate at 400 rpm. Sodium chloride (105 mM), potassium chloride (2.95 mM), magnesium chloride (4.62 mM), calcium chloride (2.34 mM), sodium carbonate (56.6 mM), disodium hydrogen phosphate dihydrate (0.337 mM), D-glucose (3.33 mM), L-ascorbic acid (1.14 mM), and bovine serum albumin (0.15 g) were sequentially added and allowed to dissolve completely. The pH was then adjusted to 7.35 ± 0.05 using concentrated hydrochloric acid. The final solution was sterilized by filtration through a 0.2 μm pore filter (Corning, supplied by Merck) to prevent bacterial contamination.

Fifteen milligrams of 5×5 μm micropatterned PLGA film were transferred into a sterilized, sealed jar and incubated in aCSF at 37 ± 0.1 °C. At predetermined time points (7, 14, and 21 days), samples were collected and weighed, while the acidity of the incubation medium was recorded.

S3. PULCON method validation from $DMSO_2$ samples. An automated protocol based on the PULCON method was optimized using dimethyl sulfone ($DMSO_2$, 94.13 Da) as a certified reference standard. ^{1}H qNMR spectra of $DMSO_2$ standard solutions in deuterated dimethyl sulfoxide (DMSO-d_6) containing 3% TFA were acquired at 298 K using a 400 MHz Avance III spectrometer. Standard solutions were prepared over a concentration range of 0.5–70 mM. PULCON-based concentration values were calculated and compared with gravimetrically determined concentrations to generate a calibration curve using the least-squares regression method.

S4. PULCON method for PLGA mass quantification. The PULCON method was validated for poly(lactide-co-glycolide) (PLGA) quantification following a partially modified protocol previously described by the authors [1]. PLGA samples were prepared at three concentrations, namely 2.84, 28.4, and 141 μM (corresponding to 1, 10, and 50 mM of repetitive units for a 38 – 54 kDa PLGA, respectively) in DMSO-d_6 containing 3% TFA, and each concentration was prepared in triplicate. ^{1}H qNMR spectra were acquired using a Bruker Avance III 400 MHz spectrometer.

S5. Erosion of micropatterned PLGA films – mass loss via qNMR. First, the PVA microlayer was removed from μMESH (n = 10) by carefully rinsing, drying, and transferring the remaining PLGA micronetwork samples into vials containing 2 mL of DI water or 0.1 M PBS pH 7.4. The vials were incubated at 37 ± 0.1 °C. At predetermined intervals, namely 0, 7, 14, 30, and 60 days, samples were collected and centrifuged to separate any soluble degradation products (12,700 rpm, 4 °C, 30 min). The supernatant was discarded, the pellet was resuspended in fresh DI water, and a second centrifugation was performed. The resulting pellets were freeze-dried, and the lyophilized samples were dissolved in a known volume of 3% trifluoroacetic acid (TFA) in DMSO-d_6 and then loaded into 3 mm disposable SampleJet tubes. All NMR spectra were recorded on a Bruker Avance III 400 MHz spectrometer. Quantitative analyses were performed automatically using 16 scans,

65536 data points, and an inter-pulse delay of 40 seconds over a spectrum of 20.49 ppm (offset at 6.17 ppm). Spectra were manually phased and automatically baseline corrected.

S6. Analysis of the erosion profiles – mass loss. Mass loss kinetics were analyzed by plotting the remaining PLGA mass as a function of time on a semilogarithmic scale to linearize exponential decay behavior (**Figure S6A-C**). The decay portion of each remaining mass curve was identified and fitted by least-squares regression according to the method described by Kenley et al. [2], using the general relationship

$$mass\ (t) = mass\ (0) * \ e^{-k_{ML}\,t} \tag{S1}$$

where the slope of each fit was taken as the decay constant k_{ML}. The k_{ML} values were determined for each configuration and incubation medium.

S7. Gel permeation chromatography. Gel permeation chromatography (GPC) analyses were performed using the same experimental conditions described in the main Methods section. Briefly, lyophilized samples were dissolved in N,N-dimethylformamide (DMF) containing 0.1% LiBr at 50 °C and analyzed using an integrated OMNISEC system (Malvern Panalytical Ltd., UK) equipped with a D6000M and a D2500 column (10 and 6 µm particle size, both 300 × 8 mm) and a triple detection setup consisting of refractive index, viscometer, and dual-angle light scattering detector (15° and 90°). DMF containing 0.1% LiBr was used as the eluent at 50 °C, with a flow rate of 1 mL/min.

S8. Analysis of the degradation profiles – change in molecular weight. The same kinetic analysis used to characterize mass loss was also applied to determine the degradation rate. Weight-average molecular weight values were plotted as a function of the incubation time on both linear (**Figure 3A-B**) and semilogarithmic scales (**Figure S7A-B**). For all tested micropatterned PLGA film configurations, pseudo-first-order kinetics were observed in both media. The slope of the curve fitting each MW decay curve was determined, corresponding to the observed rate constant (k_{DEG}) for PLGA degradation, calculated according to the following equation:

$$M_W(t) = M_W(0) * \ e^{-k_{DEG}\,t} \tag{S2}$$

Molecular weight data were analyzed up to 21 days of incubation, as values at later time points fell below the resolution limits of the GPC system.

S9. Geometric Correlation Analysis. To further evaluate the role of individual geometric parameters, additional correlation analyses were performed between μMESH features, namely filament width (w) and opening size (a), and the measured degradation and erosion (k_{ML}, k_{DEG}) and release parameters. Pearson correlation coefficients were calculated to assess the strength and statistical significance of these relationships.

S10. High-magnification Scanning Electron Microscopy analysis. Scanning Electron Microscopy (SEM) imaging was performed as described in the main manuscript. Briefly, PLGA samples were collected at the indicated time points, dried, and sputter-coated with a thin conductive layer before imaging. Additional high-magnification images were acquired under the same instrumental conditions to better resolve local morphological features, including filament thinning and structural rearrangement during degradation. Specifically, 20×20 μMESH and FLAT samples were imaged after 30 and 60 days of incubation in PBS, respectively.

S11. Drug loading and release from μMESH. A model drug, docetaxel (DTX), was loaded into each μMESH configuration and the FLAT control to evaluate the influence of geometry on pharmacological performance. Because each configuration retained a different total amount of polymer, the DTX quantity was adjusted to maintain a constant polymer-to-drug mass ratio across all samples, thereby ensuring comparable drug distribution and interaction, as described in the main manuscript.

As higher amounts of DTX were needed, chloroform ($CHCl_3$) was selected as the organic solvent due to the higher solubility of the drug in $CHCl_3$ than in acetonitrile (ACN), as previously observed [3], whereas PLGA is soluble in both solvents. To assess whether the use of $CHCl_3$ could affect the structural and pharmacological properties of the system, additional experiments were performed using the 20×20 μMESH configuration. Samples were prepared using both solvents, loaded with 1 mg of DTX, and compared in terms of morphology, drug loading, and release behavior.

Following this validation, all configurations were loaded with DTX using the same polymer-to-drug mass ratio. Drug release studies were then carried out by incubating the samples under the conditions described in the main manuscript, and the amount of DTX released over time was quantified.

S12. Micropatterned PLGA films' interaction with brain tissue. Prior to decalcification and sectioning, the explanted skulls and brains were visually inspected and imaged using a digital camera to assess gross morphology.

Supporting Results

S1. Medium acidification during full µMESH degradation in a Float-A-Lyzer® G2 dialysis device. Dual-compartment µMESH samples were placed in the Flot-A-Lyzer device without prior removal of the PVA layer. The pH variation shown in **Figure S1** is consistent with the data presented in **Figure 2B**. However, it should be noted that the latter were obtained using a sealed jar configuration, in which pre-washed µMESH samples (i.e., without the PVA microlayer) were incubated in a fixed volume of 50 mL.

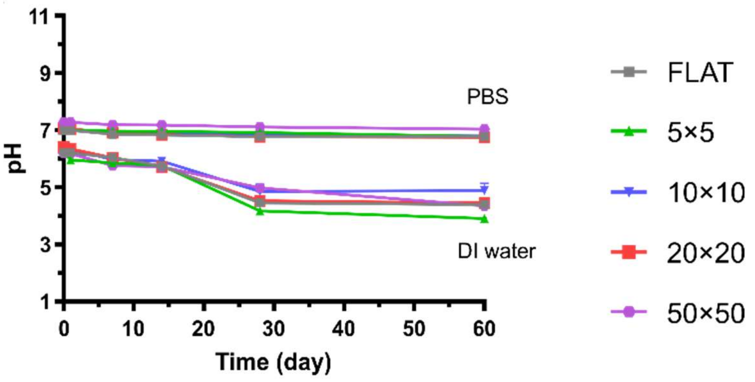

Figure S1. Change in pH vs. time for full µMESH samples and FLAT in DI water and PBS.

S2. Incubation in artificial cerebrospinal fluid. To more accurately recapitulate in vivo conditions, the micropatterned PLGA film erosion was also evaluated in artificial cerebrospinal fluid (aCSF). The resulting mass-loss profile closely matched that observed during incubation in PBS and was consistent with previously reported findings [4]. Moreover, no measurable acidification

of the aCSF medium occurred. PBS and aCSF exhibit similar osmolarity values (~300 mOsm/L [5,6]), indicating comparable salt concentrations that can moderate pH changes and influence the rate of polymer chain scission. The corresponding results are summarized in **Figure S2**.

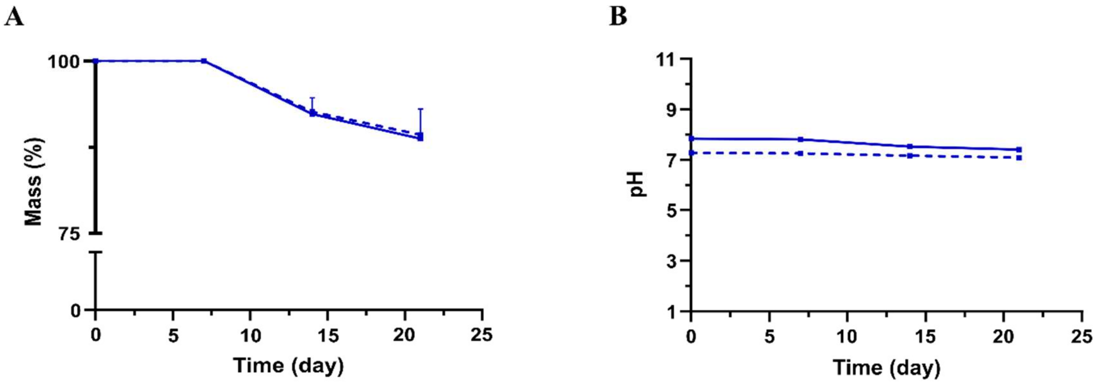

Figure S2. **(A)** Mass loss vs. time in PBS (dashed line) and aCSF (solid line) for 5×5 micropatterned PLGA film. **(B)** Change in pH vs. time in PBS and aCSF for 5×5 micropatterned PLGA film.

S3. PULCON method validation from $DMSO_2$ samples. The PULCON calibration curve obtained from $DMSO_2$ showed a strong positive linear between gravimetrically determined concentrations (c) and PULCON-derived concentrations (y) over the range of 0.5 – 70 mM correlation ($y = mc + y_0$ with $m = 0.9666$ and $y_0 = 0.1992$ – $R^2 = 0.9991$; **Figure S3A, B**). Within this range, the mean relative error and relative standard deviation (RSD) were observed to be smaller than 4% and 5%, respectively (**Figure S3C**). At a lower concentration (0.1 mM), the difference between c and y increased to 10%; therefore, to balance the speed and accuracy of the analysis, a concentration of 0.5 mM was chosen as the Limit of Quantification (LOQ) for further analyses.

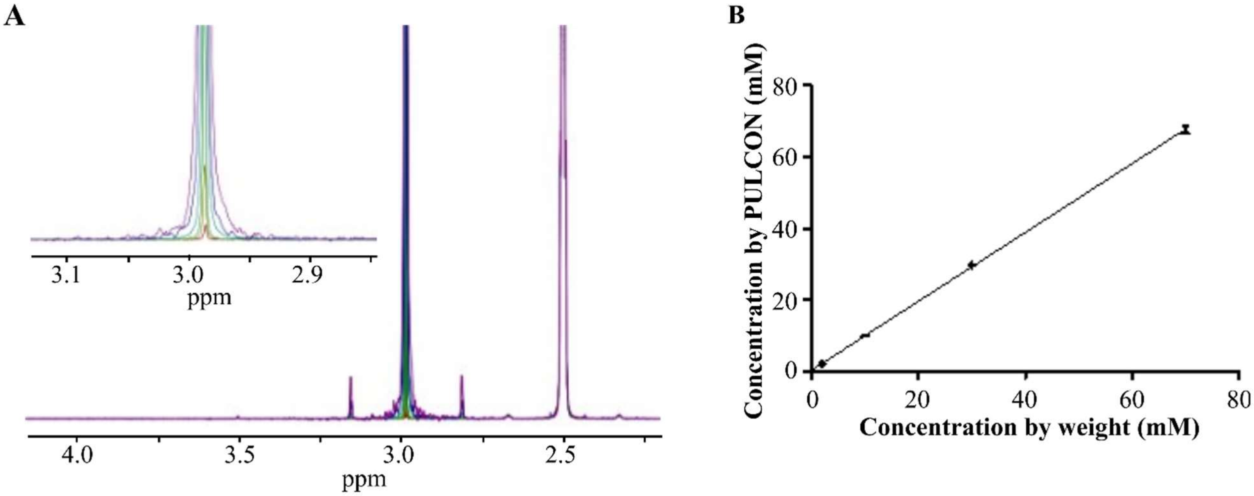

C

Weighting (mM)	PULCON (mM)	Colour of the line	Mean relative error (%)	Relative standard deviation (%)
$1.00\times10^{-1} \pm 2.89\times10^{-3}$	$1.10\times10^{-1} \pm 1.78\times10^{-4}$	Red	10.00	3.15
$5.00\times10^{-1} \pm 1.44\times10^{-2}$	$4.96\times10^{-1} \pm 8.02\times10^{-4}$	Yellow	-0.80	2.77
$2.00\times10^{0} \pm 4.91\times10^{-2}$	$2.00\times10^{0} \pm 3.23\times10^{-3}$	Green	-0.08	4.45
$1.00\times10^{1} \pm 1.04\times10^{-1}$	$9.89\times10^{0} \pm 1.60\times10^{-2}$	Light blue	-1.16	2.46
$3.00\times10^{1} \pm 8.73\times10^{-2}$	$2.97\times10^{1} \pm 4.80\times10^{-2}$	Purple	-0.94	1.03
$7.00\times10^{1} \pm 5.70\times10^{-2}$	$6.76\times10^{1} \pm 1.09\times10^{-1}$	Fuchsia	-3.38	2.68

Figure S3. Validation of the PULCON method using $DMSO_2$ standard samples. (A) ^{1}H qNMR superimposed spectra generated on a 400 MHz Avance III spectrometer, at 298 K, for dimethyl sulfone ($DMSO_2$) standard solutions in deuterated DMSO-d_6 + 3% TFA with concentrations ranging from 0.5 to 70 mM. **(B)** Calibration curve obtained by plotting the $DMSO_2$ concentrations measured by PULCON method (y) against those calculated by direct weighting (c) within the range of concentration 0.5 –70 mM. A least-squares linear regression method was used to fit the data with the function y = 0.9666 c + 0.1992 (R^2 = 0.9991). **(C)** A direct comparison between the concentrations of $DMSO_2$ obtained by weighing (first column) and measured by PULCON (second column). A color legend listed in the third column reports the color of the line in the superimposed spectra per $DMSO_2$ concentrations. The fourth and fifth columns list the mean relative error and relative standard deviation, respectively. Data are reported as mean ± absolute uncertainty for n = 3 independent repetitions. The largest error occurs at the lowest concentration of $DMSO_2$ (0.1 mM).

S4. PULCON method for PLGA mass quantification. For the spectral acquisition, both the methine group (-CH) at 5.05–5.30 ppm and the methyl group ($-CH_3$) at 1.35–1.55 ppm resonance

of PLGA were considered for quantification (**Figure S4A**). A strong linear correlation was observed between the gravimetrically determined PLGA concentrations (first column) and PULCON-derived values for both the methine and methyl signals (second and fourth columns, respectively; **Figure S4B**). Across the tested concentration range, the mean relative error was below 10.5%, confirming the accuracy of the PULCON-based quantification.

A

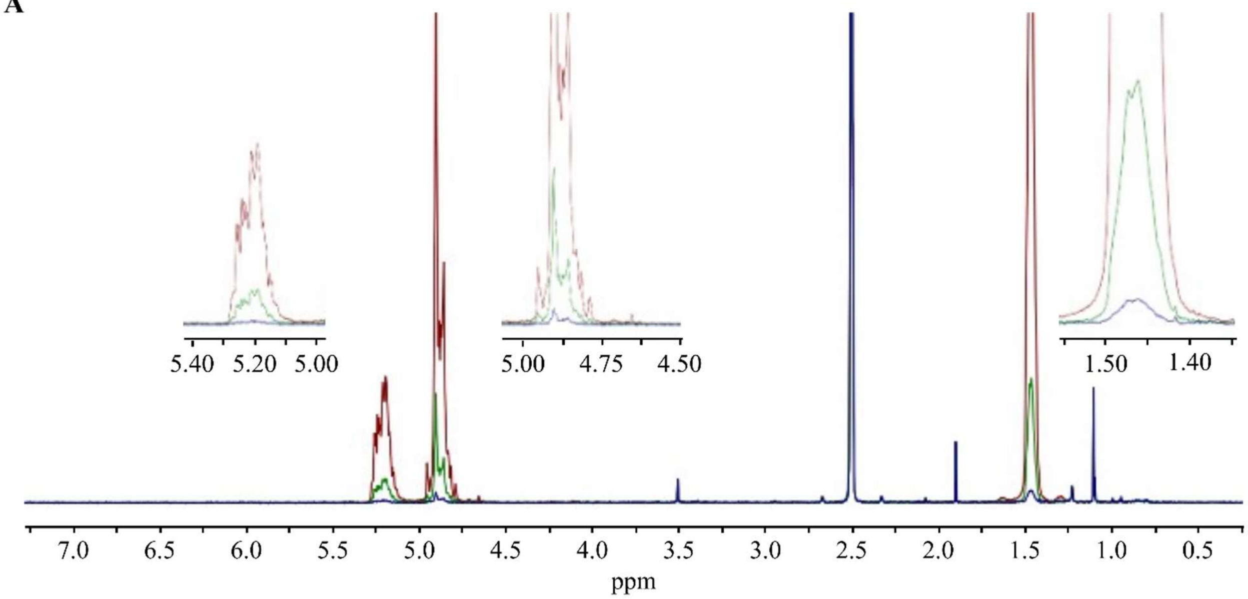

B

	Methine group (-CH) at 5.05 – 5.30 ppm		Methyl group ($-CH_3$) at 1.35 – 1.55 ppm	
**Weighting (µM)**	**PULCON (µM)**	**Mean relative error (%)**	**PULCON (µM)**	**Mean relative error (%)**
$2.84\times10^{0} \pm 2.91\times10^{-3}$	$3.03\times10^{0} \pm 4.90\times10^{-3}$	6.72	$3.00\times10^{0} \pm 4.85\times10^{-3}$	5.75
$2.84\times10^{1} \pm 2.24\times10^{-2}$	$3.11\times10^{1} \pm 5.03\times10^{-2}$	9.68	$3.13\times10^{1} \pm 5.06\times10^{-2}$	10.32
$1.41\times10^{2} \pm 1.12\times10^{-0}$	$1.37\times10^{2} \pm 2.22\times10^{-1}$	-2.94	$1.37\times10^{2} \pm 2.23\times10^{-1}$	-2.81

Figure S4. Mass quantification by ^{1}H qNMR. (A) ^{1}H qNMR superimposed spectra generated on a 400 MHz Avance III spectrometer, at 298 K, for PLGA solutions in deuterated DMSO-d_6. **(B)** A direct comparison between concentrations of PLGA obtained by weight (first column) and measured by PULCON considering the methine group (second column) and considering the methyl group (fourth column). The third and fifth columns list the mean relative errors of data measured by PULCON on methine and methyl groups, respectively. Data are reported as mean ± absolute uncertainty for n = 3 independent repetitions).

S5. Erosion of micropatterned PLGA films – mass loss via qNMR. While gravimetric mass-loss experiments focused on micropatterned PLGA films, this analysis was conducted on µMESH systems – 5×5 mm in size –, which include a PVA microlayer and distinct PLGA masses depending on microgeometry. The number of µMESH incubated for each condition was increased to match the instrument sensitivity. At predefined time points, samples were collected and the remaining PVA and PLGA were quantified using a PULCON-based qNMR protocol (**Supporting Information 3**). Quantitative spectra were acquired for all µMESH configurations and FLAT controls in both incubation media. As previously reported, the PVA microlayer dissolved rapidly, with ~98% mass loss within the first hour [7]. The remaining PLGA mass was quantified by integrating the methine (–CH–) signal at 5.05–5.30 ppm (**Figure S4**), which is well isolated from neighboring resonances (**Figure S5A**).

Figure S5B and **S5C** illustrate the evolution of the polymer mass over time, with parametric curves originating from each specific initial mass. In both PBS and DI water, FLAT samples exhibited the largest overall mass loss over the 60-day period. In PBS, the average mass loss rate, calculated as the ratio between the total mass loss and the duration of the observation period, was 3 µg/day for FLAT, followed by 2.25 µg/day for 5×5 µMESH, 1.67 µg/day for 10×10 µMESH, 0.83 µg/day for 50×50 µMESH, and 0.67 µg/day for 20×20 µMESH. Notably, the mass-loss rate of the 50×50 µMESH differed substantially from that inferred from gravimetric erosion data (**Figure 2**), likely reflecting differences in the absolute PLGA mass associated with each µMESH geometry and their corresponding S_a/V ratios.

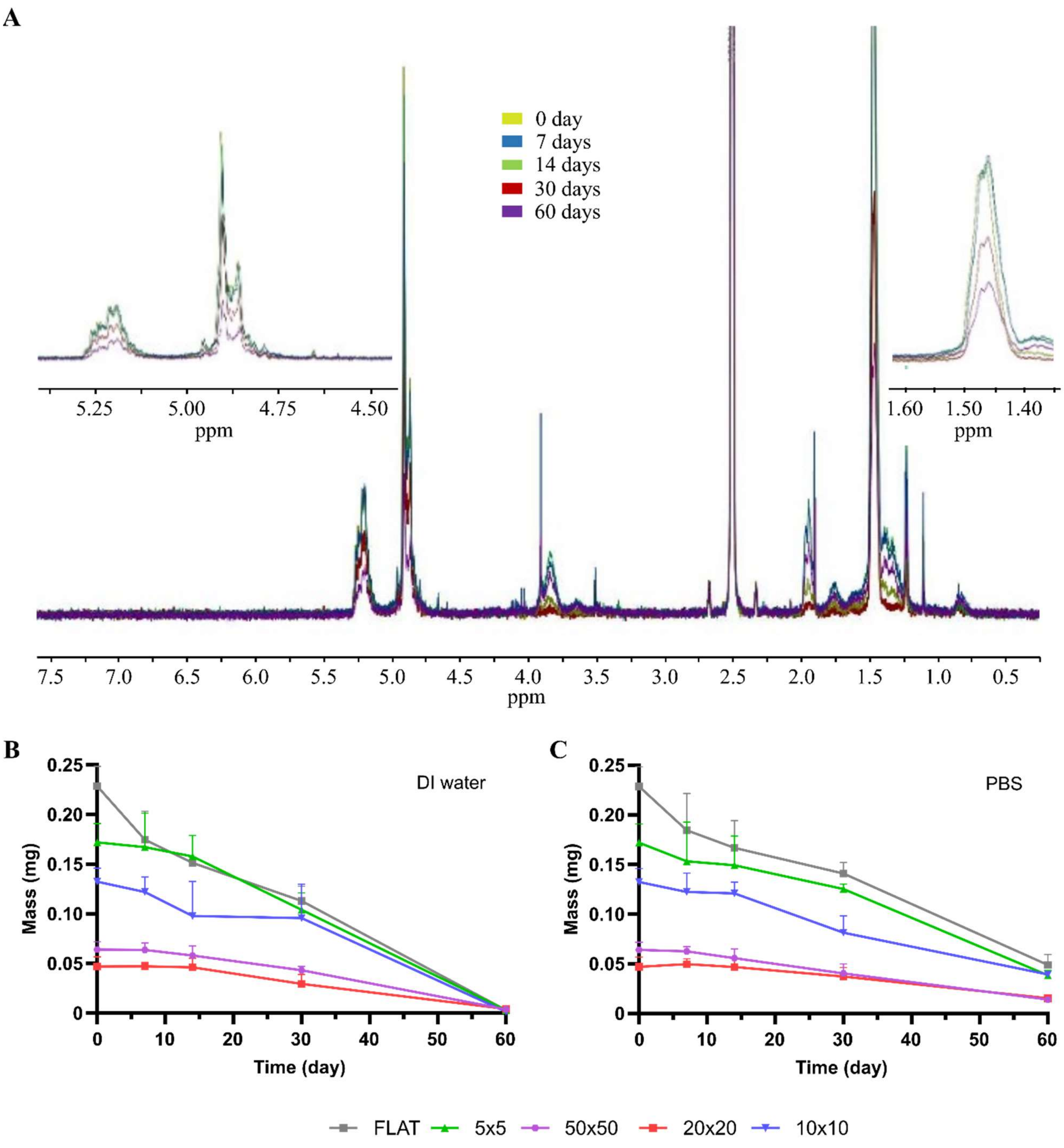

Figure S5. Erosion of micropatterned PLGA films – mass loss via qNMR. (A) ^{1}H *q*NMR spectra of a 20×20 µMESH incubated in PBS at predetermined time points, over a 60-day observation period. **(B)** Mass loss vs. time in PBS for all tested µMESH configurations and FLAT. **(C)** Mass loss vs. time in DI water for all tested µMESH configurations and FLAT.

S6. Analysis of the erosion profiles – mass loss. In PBS, semilogarithmic plots of mass loss (**Figure S6A**) revealed an initial phase with a near-zero slope, confirming the negligible mass loss. After approximately 21 days, exponential mass loss became apparent for all configurations. The decay phase was fitted using least-squares regression (**Figure S6B**), and the corresponding erosion rate constants (k_{ML}) are reported in **Table S6E**. In the buffered medium, k_{ML} showed a strong and nearly linear correlation with the parameter S_a/V, with higher S_a/V values associated with slower mass-loss rates, highlighting the key role of geometry in modulating the erosion kinetics (**Figure 2F**).

In contrast, the behavior in DI water did not allow a comparable geometry-dependent trend to be resolved. The semilogarithmic plot (**Figure S6C**) shows an earlier onset of the exponential decay phase, occurring around day 14 due to the faster mass loss. For the FLAT, 5×5, and 10×10 micropatterned PLGA film configurations, the trendlines in the semilogarithmic plots end abruptly because complete mass loss occurred by day 60. As expected, the corresponding least-squares fitting (**Figure S6D**) confirmed a faster erosion in DI water, with absolute k_{ML} values consistently higher than those measured in PBS for each geometry (**Figure S6E**), due to higher acidification and hydrolysis rates. The micropatterned films exhibited comparable k_{ML} values, whereas FLAT showed a faster rate of erosion. However, correlation analysis yielded a Pearson coefficient of r = 0.76 with a p value of ~0.14, indicating that erosion kinetics in unbuffered medium did not show any statistically significant dependence on S_a/V ratio (**Figure 2F**).

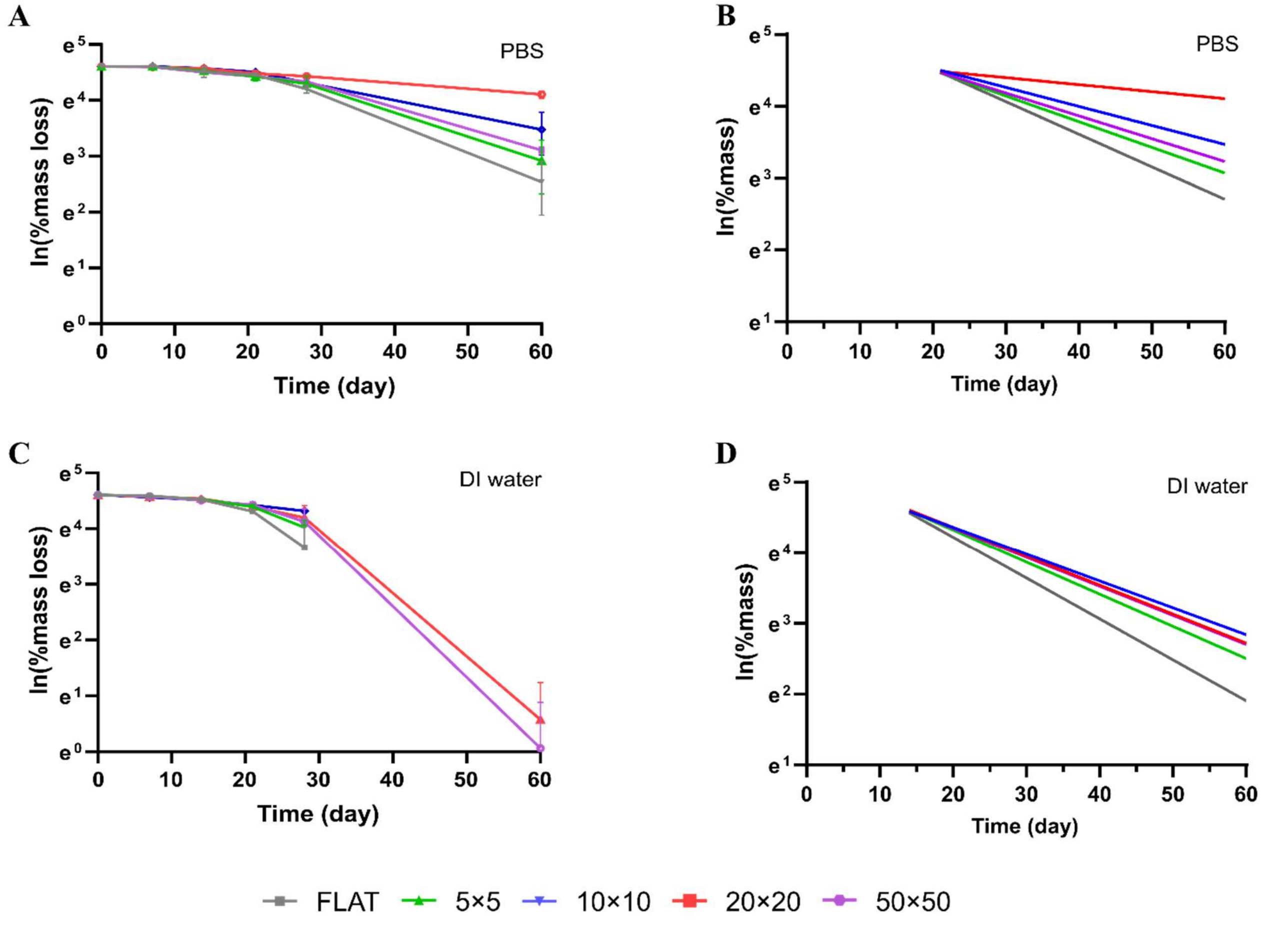

E

	PBS			DI water		
	R^2	k_{ML}	95% CI	R^2	k_{ML}	95% CI
**FLAT**	0.915	-0.0116	-0.02478 to -0.01571	0.963	-0.0252	-0.05941 to -0.00759
**5 × 5**	0.926	-0.0104	-0.02378 to -0.009027	0.944	-0.0198	-0.05464 to -0.00394
**10 × 10**	0.940	-0.0082	-0.01875 to 0.006568	0.890	-0.0165	-0.06872 to -0.00120
**20 × 20**	0.995	-0.0041	-0.005559 to -0.002973	0.935	-0.0178	-0.05236 to -0.00270
**50 × 50**	0.918	-0.0098	-0.01774 to -0.009771	0.925	-0.0179	-0.05686 to -0.00179

Figure S6. **(A)** Semilogarithmic plot of mass loss versus time in PBS for all tested micropatterned PLGA film configurations and FLAT. **(B)** Least-squares fitting of the decay phase data in PBS. **(C)** Semilogarithmic plot of mass loss versus time in DI water. **(D)** Least-squares fitting of the decay phase data in DI water. **(E)** Table reporting R^2, the slope values, and the corresponding 95% confidence intervals for each distinct fitting.

S7. Gel permeation chromatography. Representative GPC chromatograms of 10×10 μMESH at time 0 and after 7 and 21 days of incubation in DI water are shown in **Figure S7**. Elugrams were plotted as normalized refractive index (RI) signal versus elution volumes to enable direct comparison of molecular weight distribution profiles independent of sample concentration. At all time points, a single, symmetric peak was observed. With increasing incubation time, the PLGA peak progressively shifted toward longer elution times, indicating a decrease in molecular weight due to hydrolytic chain scission. In parallel, an increase in the signal corresponding to the total permeation region was observed, reflecting the accumulation of low-molecular-weight oligomeric species. At later time points, the PLGA peak approached the total permeation limit of the columns, with polymer fragments becoming too small to be resolved.

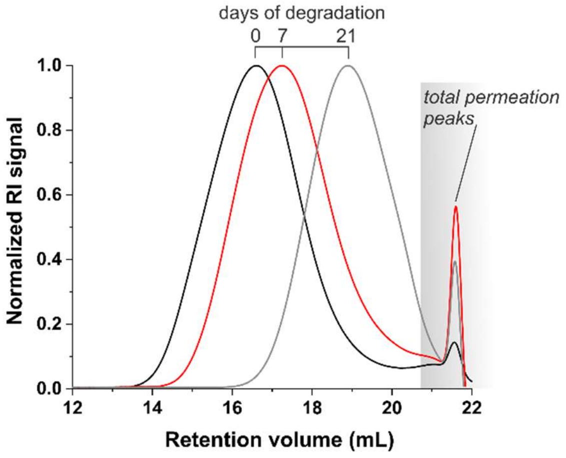

Figure S7. Representative GPC chromatograms showing normalized RI signal versus elution volume of 10×10 μMESH before incubation (t=0) and after 7 and 21 days of incubation in DI water.

S8. Analysis of the degradation profiles – change in molecular weight. All micropatterned PLGA film configurations exhibited pseudo-first-order decay in both PBS and DI water (**Figure S8A,B**). The resulting values for degradation rate constants, k_{DEG}, are reported in **Figure 3C** and listed in the Table in **Figure S8C**. The degradation mechanism proceeded similarly across all configurations in both PBS and DI water, with the only exception of 20×20 micropatterned PLGA film.

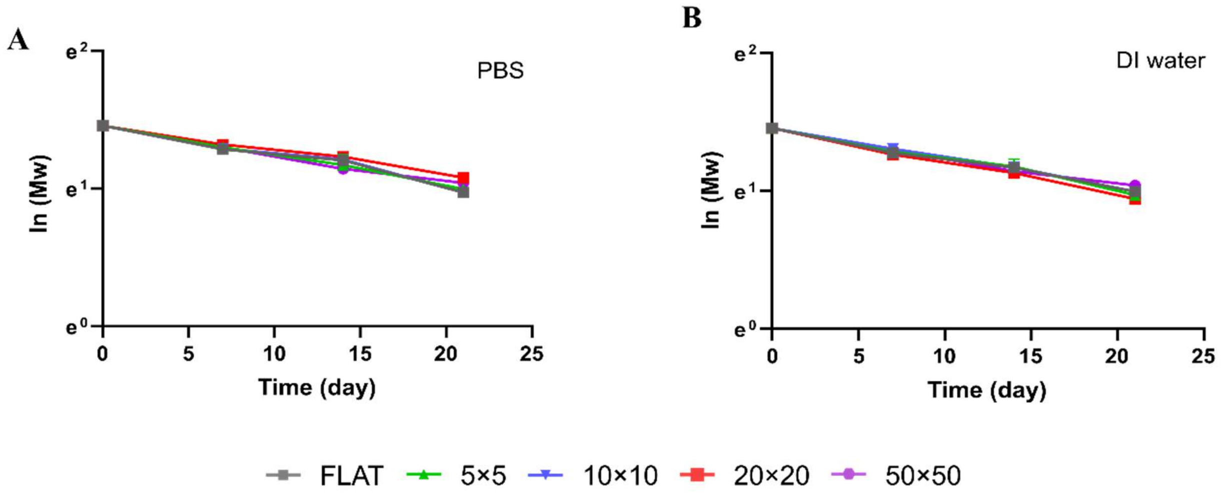

C

	PBS			DI water		
	R^2	k_{DEG}	95% CI	R^2	k_{DEG}	95% CI
**FLAT**	0.97	-0.082	-0.1009 to -0.06378	0.98	-0.087	-0.1073 to -0.06591
**5 × 5**	0.95	-0.078	-0.1028 to -0.05265	0.95	-0.077	-0.09243 to -0.06146
**10 × 10**	0.96	-0.068	-0.08734 to -0.04773	0. 90	-0.079	-0.09912 to - 0.05976
**20 × 20**	0.97	-0.055	-0.07603 to -0.03352	0.97	-0.077	-0.09890 to -0.05587
**50 × 50**	0.98	-0.067	-0.09*940 to0.03419	0.95	-0.071	-0.08780 to -0.05342

Figure S8. **(A–B)** Semi-logarithmic plot of $\overline{\mathrm{M}}_\mathrm{w}$ decay over time in PBS and DI water. © Table reporting R^2, the slope values, and the corresponding 95% confidence intervals for each distinct fitting.

S9. Geometric Correlation Analysis. The results of the correlation analyses are presented in **Figure S9**. Scatter plots of k_{ML} and k_{DEG} as a function of filament width (w) and opening size (a) are shown in **Figure S9A–D**, while the corresponding correlations for cumulative drug release (%) at day 1 and day 60 are reported in **Figure S9E,F**. Pearson correlation coefficients (r) and coefficients of determination (R^2) for all regression analyses are summarized in the table in **Figure S9G**, which also includes the values for correlation with the S_a/V ratio presented in the main text. While a weak trend was observed for w, with smaller values generally associated with lower rate constants, these correlations were not statistically significant. No meaningful correlation was

observed with a. Overall, these results indicate that neither parameter alone provides a consistent or predictive description of the observed behavior.

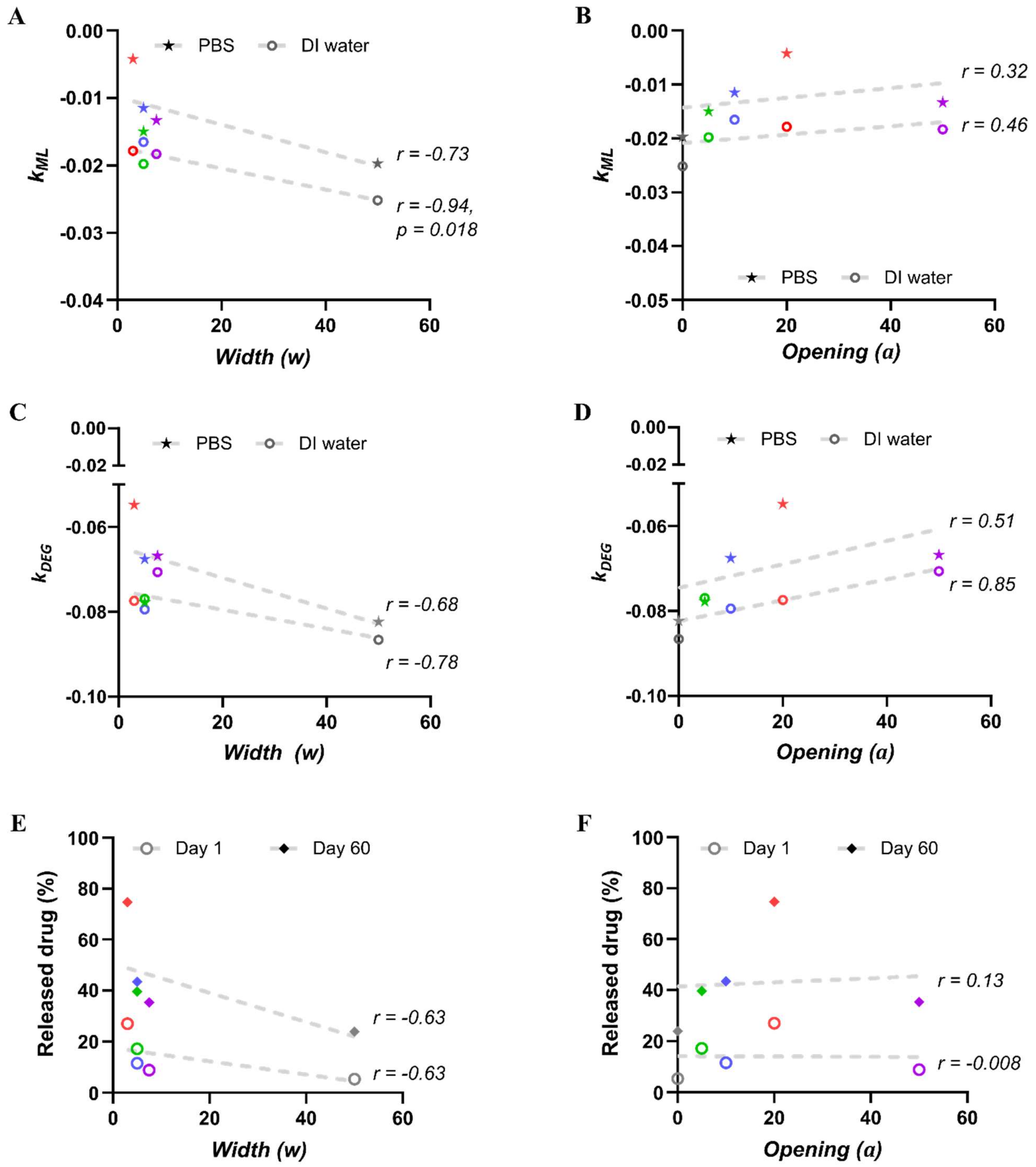

G

k_{ML}	$r_{Sa/V}$	$R^2_{Sa/V}$	r_w	R^2_w	r_a	R^2_a
PBS	0.99	0.97	-0.73	0.53	0.32	0.10
DI water	0.76	0.58	-0.94	0.88	0.46	0.21

k_{DEG}	$r_{Sa/V}$	$R^2_{Sa/V}$	r_w	R^2_w	r_a	R^2_a
PBS	0.92	0.84	-0.67	0.46	0.51	0.26
DI water	0.46	0.22	-0.78	0.61	0.85	0.72

Released drug (%)	$r_{Sa/V}$	$R^2_{Sa/V}$	r_w	R^2_w	r_a	R^2_a
1 day	0.92	0.74	-0.63	0.35	-0.0083	0.00022
60 days	0.97	0.89	-0.63	0.34	0.15	0.0078

Figure S9. Correlation analysis between geometric parameters and degradation, erosion, and drug release behavior. Scatter plots of k_{ML} (**A–B**) and (**C–D**) k_{DEG} as a function of filament width (w) and opening size (a) in PBS and DI water for all PLGA film configurations, including FLAT. (**E–F**) Scatter plots of cumulative DTX release at day 1 and day 60 as a function of w and a. **(G)** Table summarizing Pearson correlation coefficients (r) and coefficients of determination (R^2) for all regression analyses. Results of correlations with S_a/V are reported in the main text.

S10. High-magnification Scanning Electron Microscopy analysis. High-magnification images were acquired to further illustrate the morphological changes described in the main text. As shown in **Figure S10A**, 20×20 µMESH exhibits progressive thinning of polymer filaments during erosion, whereas the FLAT configuration (**Figure S10B)** undergoes structural collapse into a dense and tortuous morphology.

A

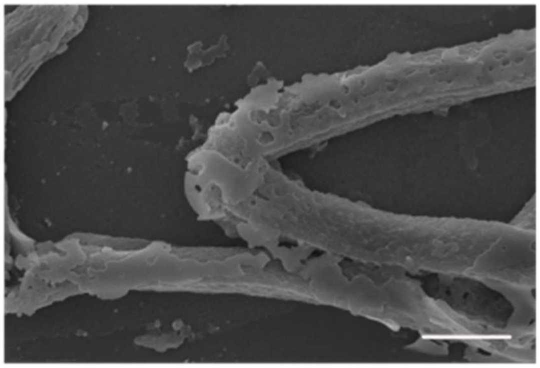

B

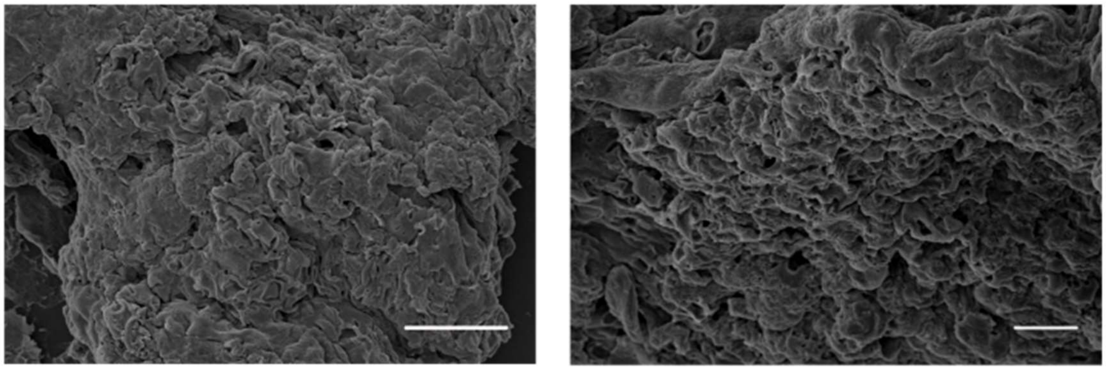

Figure S10. High-magnification SEM analysis of morphological evolution in µMESH and FLAT configurations. (A) Representative SEM image of 20×20 µMESH strands during erosion, showing progressive thinning of the PLGA filaments due to surface erosion and exfoliation of outer polymer layers (scale bar: 5 µm; magnification: ×4300). **(B)** Representative SEM image of the FLAT configuration, illustrating the loss of the initial planar film structure and its transformation into a dense, tortuous polymer aggregate (left: scale bar: 50 µm; magnification: ×500; right: scale bar: 20 µm; magnification: ×800).

S11. Drug loading and release from µMESH. To evaluate the effect of solvent choice, 20×20 µMESH loaded with DTX were prepared using ACN and $CHCl_3$ and compared. As shown in **Figure S11A,B**, no significant differences in morphology or characteristic dimensions were observed, and no formation of voids or increased surface roughness was detected. Moreover, comparable drug entrapment and release rates were documented (**Figure S11C,D**), demonstrating that the use of $CHCl_3$ does not affect the pharmacological performance of the system.

Based on these results, $CHCl_3$ was used for all subsequent experiments to ensure complete solubilization of DTX. The total amount of drug contained in a single 5×5 mm µMESH is reported in **Figure 5A**, confirming that drug loading scales with the amount of polymer used in each configuration while maintaining a constant polymer-to-drug ratio.

The cumulative amount of drug released over time is shown in **Figure S11E**. As expected, configurations with higher initial drug loading exhibited proportionally higher cumulative release. In contrast, the FLAT configuration displayed reduced drug release despite comparable surface area, likely due to the absence of microgeometry and the associated longer effective diffusion pathway. Indeed, although FLAT and 10×10 µMESH share the same S_a value (50.1×10^6 µm^2),

their pharmacological performance differs, highlighting the role of micropatterning in shortening diffusion pathways and facilitating drug transport.

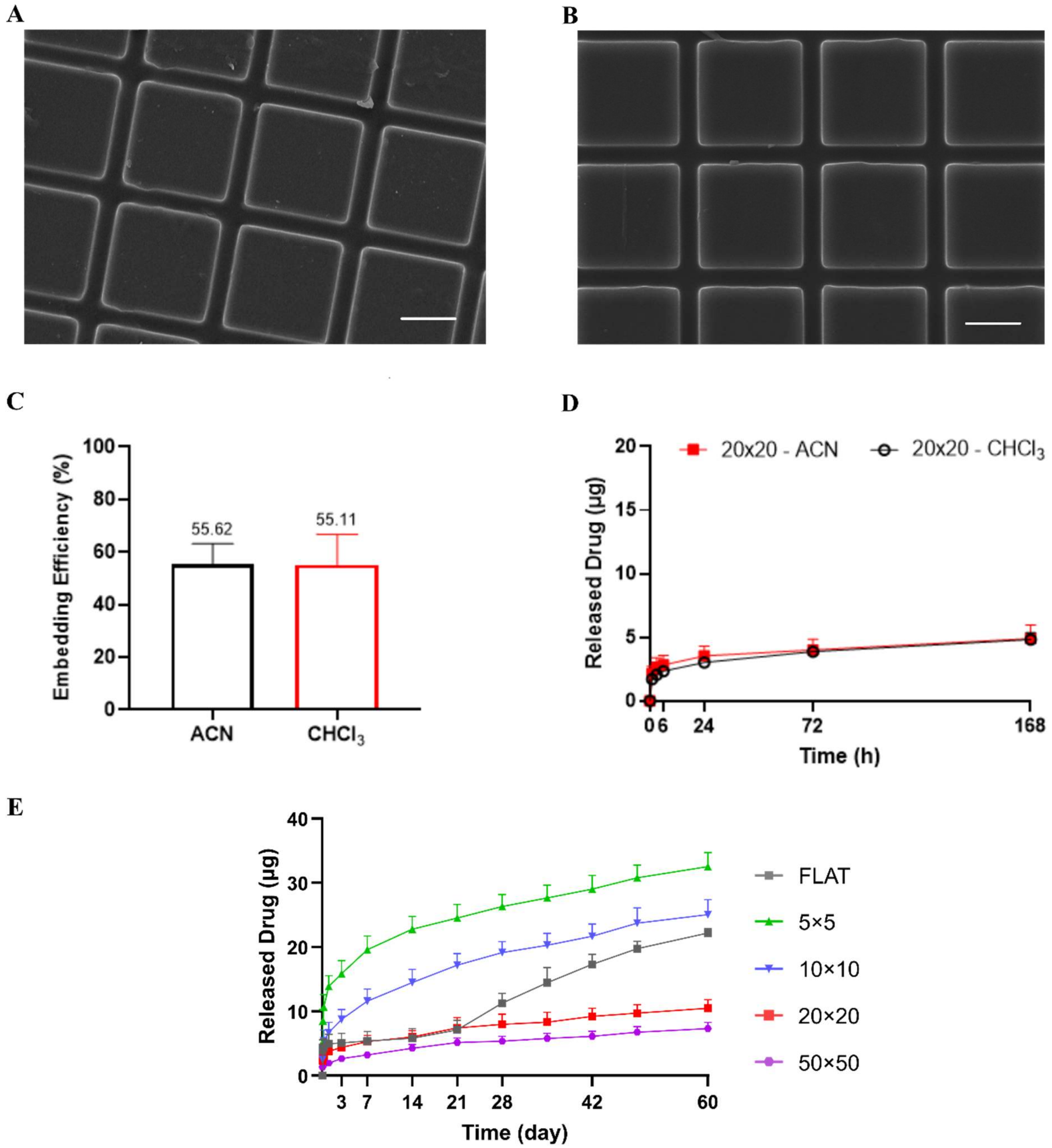

Figure S11. Effect of solvent choice on µMESH morphology, drug loading, and release behavior. (A-B) SEM images of 20×20 µMESH loaded with 1 mg of DTX and fabricated using ACN and $CHCl_3$, respectively. **(C)** Drug entrapment efficiency (%) in µMESH. **(D)** Cumulative drug release profiles over 7 days. **(E)** Cumulative release of DTX from all tested µMESH and FLAT over 2 months.

S12. Micropatterned PLGA films - interaction with brain tissue. No gross abnormalities were observed in the brain and skull, with the two columns referring to different mice.

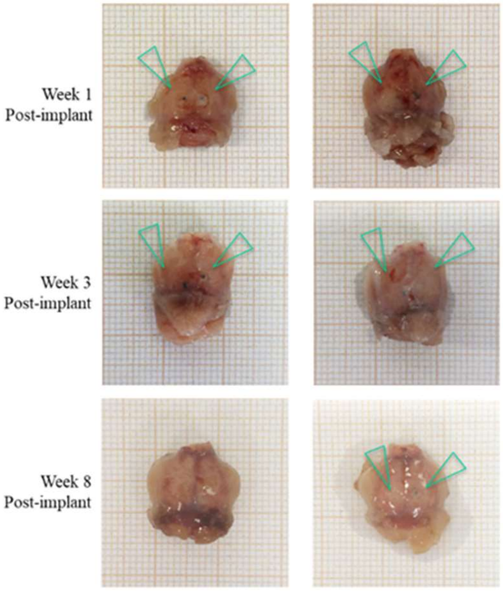

Figure S12. Macroscopic evaluation of the implant site. Representative photographic images of the brain and the overlying skull at 1-, 3-, and 8-week post-implantation, acquired prior to histological analysis (graph paper scale: 1 mm). Arrows indicate the implant site.

SUPPORTING REFERENCES

(1) Pesce, C.; Goldoni, L.; Papa, V.; Palange, A. L.; Di Mascolo, D.; Caliceti, P.; Decuzzi, P. One-Step Precise Characterization of Drug Delivery Systems by Pulcon Magnetic Resonance Spectroscopy. *Mol Pharm* **2024**, *21* (6), 2937-2948.

(2) Kenley, R. A.; Lee, M. O.; Mahoney, T. R.; Sanders, L. M. Poly (Lactide-Co-Glycolide) Decomposition Kinetics in Vivo and in Vitro. *Macromolecules* **1987**, *20* (10), 2398-2403.

(3) Di Mascolo, D.; Guerriero, I.; Pesce, C.; Spanò, R.; Palange, A. L.; Decuzzi, P. Mmesh-Enabled Sustained Delivery of Molecular and Nanoformulated Drugs for Glioblastoma Treatment. *ACS nano* **2023**, *17* (15), 14572-14585.

(4) Kou, J. H.; Emmett, C.; Shen, P.; Aswani, S.; Iwamoto, T.; Vaghefi, F.; Cain, G.; Sanders, L. Bioerosion and Biocompatibility of Poly(D,L-Lactic-Co-Glycolic Acid) Implants in Brain. *Journal of Controlled Release* **1997**, *43* (2), 123-130.

(5) An, J. H.; Su, Y.; Radman, T.; Bikson, M. Effects of Glucose and Glutamine Concentration in the Formulation of the Artificial Cerebrospinal Fluid (Acsf). *Brain Research* **2008**, *1218*, 77-86.

(6) Son, M.; Lee, Y. S.; Lee, M. J.; Park, Y.; Bae, H. R.; Lee, S. Y.; Shin, M. G.; Yang, S. Effects of Osmolality and Solutes on the Morphology of Red Blood Cells According to Three-Dimensional Refractive Index Tomography. *PLoS One* **2021**, *16* (12), e0262106.
